# Traditional Chinese medicine interventions based on meridian theory for pain relief in patients with primary dysmenorrhea: a systematic review and network meta-analysis

**DOI:** 10.3389/fmed.2024.1453609

**Published:** 2024-09-05

**Authors:** Shu-Cheng Chen, Jia-Yin Ruan, Bohan Zhang, Lok-Yi Pang, Linda Zhong, Shuang-Lan Lin, Ka-Po Wong, Hui-Xi Ouyang, Wing-Fai Yeung, Qin-Wei Fu, Bo-Qian Chen

**Affiliations:** ^1^School of Nursing, Hong Kong Polytechnic University, Kowloon, Hong Kong SAR, China; ^2^School of Biological Sciences, Nanyang Technological University, Singapore, Singapore; ^3^Department of Applied Social Sciences, The Hong Kong Polytechnic University, Kowloon, Hong Kong SAR, China; ^4^The Department of Rehabilitation Sciences, Hong Kong Polytechnic University, Kowloon, Hong Kong SAR, China; ^5^Hospital of Chengdu University of Traditional Chinese Medicine, Chengdu, China; ^6^Intensive Care Unit, Shenzhen Traditional Chinese Medicine Hospital, Guangzhou University of Chinese Medicine, Shenzhen, China

**Keywords:** primary dysmenorrhea, TCM, Chinese medicine, acupuncture, acupressure, moxibustion, *tuina*, warm needling

## Abstract

**Objective:**

To determine the comparative effects and safety of traditional Chinese medicine (TCM) interventions based on meridian theory for pain relief in patients with primary dysmenorrhea (PD).

**Methods:**

This is a systematic review with network meta-analysis. Randomized controlled trials (RCTs) comparing meridian-based TCM interventions with waitlist, placebo, western medicine, and conventional therapies for PD pain. A SUCRA was used to estimate the probability ranking for the effects of interventions.

**Results:**

57 RCTs involving 3,903 participants and 15interventions were included. Thirty-two RCTs were rated as low risk of bias. A network diagram was drawn with 105 pairs of comparisons. Compared with NSAIDs and waitlist, significantly better effects were found in acupressure [SMD = −1.51, 95%CI (−2.91, −0.12)/SMD = −2.31, 95%CI (−4.61, −0.02)], warm needling [SMD = −1.43, 95%CI (−2.68, −0.18)/SMD = −2.23, 95%CI (−4.43, −0.03)], moxibustion [SMD = −1.21, 95%CI (−1.85, −0.57)/SMD = −2.10, 95%CI (−3.95, −0.07)], and acupuncture [SMD = −1.09, 95%CI (−1.62, −0.55)/SMD = −1.89, 95%CI (−3.67, −0.11)]. No adverse events were detected.

**Conclusion:**

For PD pain, the effects of acupressure, acupuncture, warm needling, and moxibustion were superior to those of NSAIDs and waitlist. Oral contraceptive pill, electro-acupuncture, acupressure, and warm needling demonstrated higher probabilities of being better interventions. More high-quality clinical trials are needed to provide more robust evidence of this network.

**Systematic review registration:**

PROSPERO CRD42022373312.

## 1 Introduction

Primary dysmenorrhea (PD) refers to the occurrence of menstrual cramps in the lower abdomen, without any identifiable pelvic pathology, with characteristic symptoms such as lower abdominal or pelvic pain, ranging from 8 h to 72 h and typically occurring at the onset of menstrual flow (1). Other associated symptoms include low back pain, headache, diarrhea, fatigue, nausea, or vomiting (2). The prevalence of PD varies from 50 to 90% (3). The exact causes remain unknown, leading to non-targeted treatments. Risk factors for PD include early age at menarche, heavy menstrual flow, nulliparity, family history of dysmenorrhea, and stress (4).

Systematic reviews and randomized controlled trials (RCTs) have shown that non-steroidal anti-inflammatory drugs (NSAIDs) (5, 6) and hormonal regulation through oral contraception (7) are significantly more effective for pain relief than placebo, and they are often used for symptom control (8). However, some women may not always find them effective or acceptable; for example, NSAIDs have undesirable side effects (6). Non-drug, non-surgical treatments for PD include transcutaneous electric nerve stimulation, behavior modification/relaxation, acupressure, acupuncture, spinal manipulation, topical heat, vitamin E, thiamine, fish oil, and herbal medicine (9). Only 26.7% of patients with PD have no experience with complementary and alternative medicine. Diet and homeopathy are the most frequently used methods from complementary and alternative medicine. In spite of the provision of information on efficacy, safety, and costs, patients choose methods based on earlier experience (10). Despite their popularity, evidence on the effectiveness of these nonpharmacologic therapies for PD is limited and inconsistent (11, 12).

Traditional Chinese medicine (TCM) interventions based on meridian theory can be an effective alternative approach for PD. Meridian is a system of conduits through which *qi* and blood circulate, connecting the bowels, viscera, extremities, superficial, organs, and tissues and making the body an organic whole. The meridian system includes 12 regular meridians, 8 extra meridians, 12 meridian divergence, 12 meridian sinews, 12 cutaneous vessels, and 15 collateral vessels (13). Meridian-based TCM interventions include acupuncture, acupressure, *tuina*, moxibustion, cupping, auricular therapy, electro-acupuncture, and scraping. These interventions may produce analgesic effects through the regulation of the hypothalamic–pituitary–ovarian axis, modulation of the immune system, and release of endogenous opioid peptides (14). Animal experiments have demonstrated that acupuncture may facilitate the release of central and peripheral neurotransmitters, modulation of immune function, and alleviation of uterine smooth muscle spasm (15, 16). Clinical trials have indicated that acupuncture may elicit analgesia by regulating serum levels of prostaglandins and ovarian hormones, facilitating the release of peripheral β-endorphin, improving the status of uterine artery blood flow, and alleviating uterine smooth muscle spasm (14). Similarly, moxibustion may modulate endocrine hormones, immune function, and nerve factors and enhance uterine microcirculation (17).

Numerous studies have investigated the efficacy of TCM interventions based on meridian theory for dysmenorrhea, including systematic reviews, RCTs, and case series. A systematic review of RCTs on acupuncture for PD reported that acupuncture provides superior pain relief (OR: 4.99; 95% CI: 2.82–8.82; 4 RCTs; *I^2^* = 0%) and has fewer adverse events compared with NSAIDs (OR: 0.10; 95% CI: 0.02–0.44; 4 RCTs; *I^2^* = 15%); however, evidence quality was low, and further research is needed to ascertain the effectiveness of acupuncture and acupressure (18). In an RCT of 152 subjects, moxibustion was found to have sustained and superior pain-relieving effects compared with drugs 3 months post-intervention (effect size: −0.87, 95% CI: −1.32 to −0.42, *p* < 0.001) measured by the visual analogue scale (VAS) (19). Acupressure on the SP6 meridian was found to be superior to placebo acupressure in relieving PD pain symptoms based on a VAS score (−4.935; 95% CI: [−15.757, 5.887]; *p* = 0.371) (20), whereas auricular therapy was found to be more effective than analgesics based on VAS scores (OR  = 3.28, 95%CI: [1.37, 7.85], *p* = 0.008) (21). Although numerous studies have examined the effects of meridian-based TCM interventions for PD pain, no comparative analysis of their effectiveness has been conducted. Therefore, a comprehensive comparison of multiple TCM interventions based on meridian theory is required, as well as organization and collection of high-quality evidence.

Network meta-analysis (NMA) yields high-quality evidence, which can be used to evaluate the effects of multiple interventions and rank optimal intervention strategies (22). This study performed an NMA of RCTs on TCM interventions based on meridian theory for relieving pain in patients with PD, comparing the effects among interventions (TCM interventions based on meridian theory and eligible control interventions), and exploring the optimal one. The findings can provide evidence to help clinical workers and researchers understand the optimal protocol for pain management of PD.

## 2 Methods

This systematic review was reported in accordance with the Preferred Reporting Items for Systematic Reviews and Meta-analyses (PRISMA) (18), the PRISMA-2020 guidelines (19), and the extension statement for network meta-analysis (PRISMA-NMA) (20). The protocol was registered on PROSPERO (CRD42022373312).

### 2.1 Selection criteria

Studies had to fulfill the following criteria presented in PICOS tools (population, intervention, comparators, outcomes, and study design): (1) population: patients diagnosed with PD; (2) intervention: meridian-based TCM interventions including acupressure, acupuncture, moxibustion, *tuina*, electroacupuncture, warm needling, auricular therapy, and scraping therapy (Interventions should be singly used, so that their effects can be detected. Interventions related to Chinese herb, staging acupoint catgut embedment therapy, acupuncture point injection therapy, or combined TCM intervention were excluded.); (3) comparators: control group with waitlist, placebo, western medicine (e.g., NSAIDs and combined oral contraceptives), conventional therapy, or another type of meridian-based TCM interventions (Studies comparing two or more meridian-based TCM interventions were included. Usual care was excluded due to the variety of care content among different interventions and study settings.); (4) outcomes: studies that evaluated pain intensity using VAS or numerical rating scale (NRS); and (5) study design: RCTs.

### 2.2 Search strategy

We searched Ovid MEDLINE, Embase, Health Technology Assessment Database, Cochrane Central Register of Controlled Trials, Web of Science, Allied and Complementary Medicine, the China National Knowledge Infrastructure, Wanfang Data, PubMed, SinoMed, and CQVIP from database inception until 1 July 2023. The following keywords were used for our search: (massag* OR anmo OR acupress* OR *tuina* OR acupunct* OR electroacupunct* OR electro-acupunct* OR acupoint* OR meridia* OR auricular OR needl* OR moxibustion OR moxa) AND (dysmenorr* OR menstrua* pain OR period cramp OR period pain* OR menstrua* distress OR period distress OR menstrua* distress). To search the Chinese databases, we used the corresponding Chinese keywords. We did not impose any language restrictions (See Supplementary Appendix 1). We also scanned reference lists of relevant systematic reviews and clinical guidelines. Two authors (SCC and JYR) independently performed the literature search. Disagreements were resolved upon consultation and judgement by a third reviewer (WFY).

### 2.3 Data extraction

Two authors (SSC and LYP) independently screened the titles and abstracts of the retrieved studies, and the unrelated articles were removed. Thereafter, we reviewed the full texts of potentially relevant studies and extracted the data based on the selection criteria. The following data were extracted from the included RCTs in a predesigned data sheet: the first author’s name, publication year, characteristics of participants, sample size, diagnostic system, details of interventions, details of the control interventions, follow-up period, details of outcomes, and language. Basic information on the excluded studies was also extracted to provide a comprehensive reference to readers, with information on the original title, publication year, and reasons for exclusion. An additional reviewer (WFY) was consulted in the event of discrepancies between authors.

### 2.4 Quality assessment

Two authors (SCC and LYP) performed the quality evaluation of included studies independently in accordance with Cochrane Handbook for Systematic Reviews of Interventions version 6.3 (21). The risk of bias for trials was assessed using criteria in the Cochrane Risk of Bias version 2 (RoB 2) tool. We assessed potential bias related to five domains, namely, randomization process, deviations from intended interventions, missing outcome data, measurement of the outcome, and selection of the reported result. Each publication was identified as “high risk,” “low risk,” or “some concerns.” If there were any objections, they would be discussed or judged by a third researcher (WFY) to reach a consensus.

The GRADEpro Guideline Development Tool web page was employed to assess the evidence for the quality of outcome indicators including five degrading factors: risk of bias, inconsistency, indirectness, imprecision, and publication bias (23). On the basis of these variables, the software generates a GRADE rating of “high,” “moderate,” “low,” or “very low” to reflect the certainty of the reported effect. A summary of findings (SoF) table was generated for a given comparison of interventions to provide key information concerning the magnitudes of relative and absolute effects of the interventions examined, the amount of available evidence, and the quality of available evidence (21).

### 2.5 Statistical analysis

Standard mean difference (SMD) with a 95% confidence interval (CI) was used to estimate the effect sizes of NRS or VAS. Variation was expected between the original studies. To make the results more conservative, a random effects model was used rather than a fixed effects model. The statistical heterogeneity in each pairwise comparison was assessed using inconsistency index tests or Chi-square test or *I*^2^ statistic test (*I*^2^ > 30% or *p*-value <0.1 indicating inconsistency). Additionally, sensitivity analysis was conducted to verify the robustness of the results and test the source of heterogeneity in each RCT. Three-armed RCTs were transformed into two-armed pairs to clearly present the effects of comparisons. For the studies that reported the mean and standard deviation of effect changes in the interventions, we calculated the data at the endpoint of the intervention period using the current data.

An NMA with a Bayesian framework using Statistics and Data Science (STATA) software was conducted to assess the outcomes of different interventions (24). Network diagrams were used to present the results: (1) each node represents an exercise intervention; (2) the size of the node indicates the sample size of the subjects who performed this intervention; (3) if there are no line segments between each node, then indirect comparisons will be made between the nodes; if there are line segments, then direct comparisons will be made between the nodes; (4) the thickness of the line segments between the nodes indicates the original study sample size; and (5) the size of the nodes and the thickness of the line segments are positively correlated with the number (25). The surface under the cumulative ranking curve (SUCRA) is the probability each intervention has of being among the best of those in the network; the SUCRA values range from 0 to 1, with large values representing high intervention ranking probabilities (26). NMA results were visualized using forest plots. A funnel plot was generated to examine possible publication bias.

## 3 Results

### 3.1 Literature search

**Figure 1:**
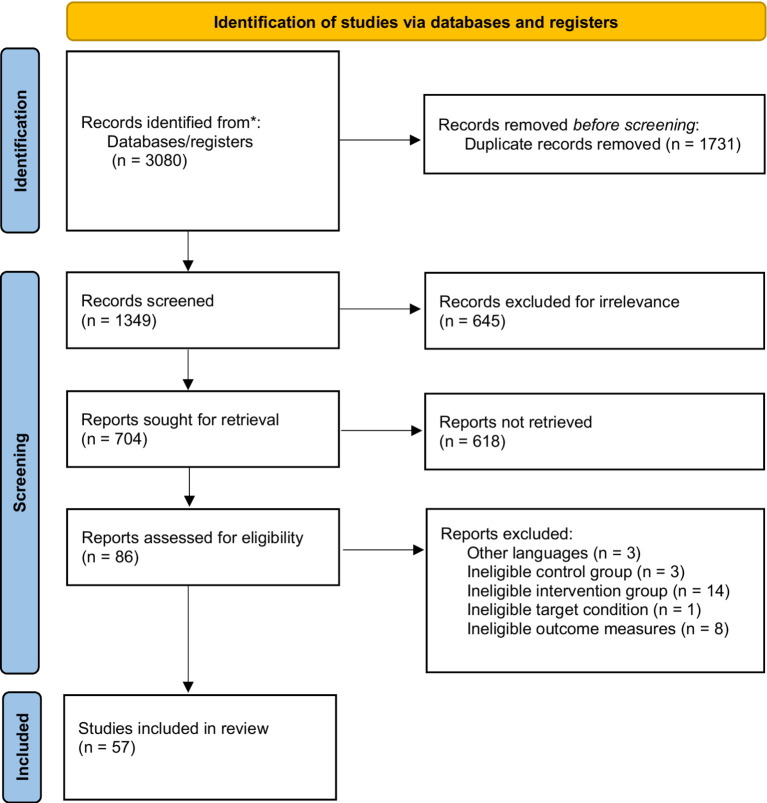
PRISMA Flow Chart.

Our search yielded a total of 3,080 potentially eligible citations, among which 704 remained after the removal of duplicates and irrelevant records. Of the remaining citations, 86 studies were obtained by reading the titles and abstracts. After the full text of these records was screened, 57 studies were included for network analysis. The PRISMA flow diagram describing the inclusion process is presented in Figure 1. Supplementary Appendix 2 shows the details of the excluded studies from full-text screening.

### 3.2 Description of included studies

As shown in Table 1, the included 57 RCTs were published between 2011 and 2022 and included 3,903 participants. The included participants’ age ranged from 14 years to 39 years. The sample size of the included RCTs was from 20 to 208. Besides the eligibility on age, disease duration, and intervention use records, most RCTs screened and included participants by using pain rating scales (57 studies), including VAS and NRS. Eight types of meridian-based TCM interventions were reported among the included RCTs, namely, acupuncture (*n* = 865) (31, 32, 35–37, 41, 44, 46, 50–56, 58, 62–65, 68, 70), acupressure (*n* = 285) (27, 28, 30, 33, 34, 71, 72, 79), auricular therapy (*n* = 35) (57), electro-acupuncture (*n* = 66) (34, 47), moxibustion (*n* = 651) (29, 38–40, 42–44, 48–50, 61, 62, 66, 67, 74, 77), scraping therapy (*n* = 30) (69), *tuina* (*n* = 57) (45, 54), and warm needling (*n* = 151) (41, 59, 60, 78). The most commonly involved meridians included conception vessel (CV), spleen meridian (SP), stomach meridian (ST), and governor vessel (GV). Four types of control interventions were reported, namely, health education (27), waitlist (35, 81), placebo (sham interventions) (28–30, 32, 33, 56, 72, 82), and conventional medicine (oral contraceptive pill and NSAIDs) (31, 36–40, 42, 43, 45–49, 51–53, 55, 57–61, 63–71, 73–80, 83). Pain outcome measures included VAS and NRS. One RCT used both VAS and NRS (29), 3 used NRS only (36, 59, 69), and the other 53 used VAS only. The treatment period ranged from 30 min to 3 months, and the follow-up period ranged from 3 h to 6 months. Twelve RCTs were published in English and 45 were in Chinese.

**Table 1:** Characteristics of the included studies.

NO.	First author (year)	Age (mean, range)	Sample size (I/C)	Inclusion criteria	Exclusion criteria	Diagnostic system	Treatment	Control	Outcome measure (pain)	Treatment/ follow-up period	Language
1	Chen (2015) (27)	NR, NR	129 (65/64)	- Women ≤25y - VAS ≥ 4	- Receiving pain medication in the recent 4 h - had a surgery for low back pain	NR	Acupressure (SP6, BL32, Lv3)	Health education	- VAS	1 m / 12 m	English
2	Dincer (2023) (28)	NR, NR	67 (34/33)	- Women ≥18y - VAS ≥ 4 - Regular menstrual period of 3-8d with an interval of 21-35d	- Having pelvic disease, operation, infection, physical or mental illness, NSAIDs allergy - receiving COC	NR	Acupressure (SP6)	Sham acupressure (SP6)	- VAS	3 m / 1 m	English
3	Gao (2015) (29)	NR, NR	56 (28/28)	- Nursing students - 17-25y - Regular menstrual cycles 21-35d and length 3-7d	- Having received hormonal therapy in the past 6 m - Receiving analgesics during the study period - With SDM, systemic or gynecologic disease	NR	Moxibustion (CV4, CV8)	Sham moxibustion (CV4, CV8)	- NRS - VAS	2 m / NR	English
4	Kashefi (2010) (30)	NR, 18–26	86 (43/43)	- Iranian single female college students - 18-28y - VAS ≥ 4 - regular menstrual cycles	- Having recent stressful events - Having history of gynecological disease or SDM - receiving pain medication before each menstrual period and 3 h after intervention	NR	Acupressure (SP6)	Sham acupressure	- VAS	2 m / NR	English
5	Kiran (2013) (31)	21, NR	35 (11/24)	- Women aged 15–40y - Severe persist pain before/during menstruation - Being referred to the outpatient clinic of obstetrics	-Having irregular menstrual cycles or SDM	- Based on self-reported severe	Acupuncture (HT7, PC6, LI4, LI10, SP6, LR3, ST36, GB26, SP15, CV19)	NSAIDs (naproxen sodium)	- VAS	3 m / 12 m	English
6	Liu (2022) (32)	NR, NR	47 (22/19)	- Women aged 18–30y with at least 1y history of PDM - VAS ≥ 4 - Regular menstrual cycle 27-32d - No hormones or centrally acting drugs in the past 6 m	- Having SDM or severe chronic diseases - Being pregnant, prepared for pregnancy/breastfeeding - Receiving acupuncture, analgesic medication in the past 3 m	- Society of Obstetricians and Gynecologists of Canada’s diagnostic criteria	Acupuncture (SP6)	Sham acupuncture (SP6)	- VAS	3 m / NR	English
7	Mirbagher-Ajorpaz (2011) (33)	22, NR	30 (15/15)	- Women aged 18-30y - Regular menstrual cycles - VAS ≥ 3	- Being pregnant - Having SDM	- Based on history and then by exclusion of probable pathologies	Acupressure (SP6)	Light touch (SP6)	- VAS	30 min / 3 h	English
8	Qorbanalipour (2018) (34)	NR, NR	64 (31/33)	- Virgin girls aged 18-26y - At least 2y of regular menstruation between 21-35d	- With SDM, history of pelvic organs surgery, itching, abnormal secretions - Using any other drugs for health problems in the past 6 m - with a history of smoking or alcohol use	- Diagnosis from a gynecologist	Electro-acupuncture (SP4, SP6)	Acupressure (SP4, SP6)	- VAS	2 m / 1 m	English
9	Shetty (2018) (35)	NR, 17–23	60 (30/30)	- Women with PDM for at least 1y - Regular menstrual cycles 28 ± 7d	- With SDM or any systemic psychiatric illness - Receiving intrauterine devices and medicine for PDM in the past 6 m	NR	Acupuncture (KI3, SP8, ST25, ST29, ST30, ST36, CV4, CV6, BL62, HT7, LI4, PC6)	Waitlist	- VAS	2 m / 1 m	English
10	Sriprasert (2015) (36)	NR, 18–35	52 (27/25)	- With PDM within the previous 3 months - NRS ≥ 5 - VMSS ≥2	- With contraindication to COC/acupuncture and abnormal gynecologic conditions - Underlying gynecologic conditions associated with DM	- Diagnosis from a gynecologist	Acupuncture (CV6, CV3, SP8, SP6)	Oral contraceptive pill	- NRS	3 m / NR	English
11	Wang (2019) (37)	NR, NR	62 (31/31)	- Women aged 18-35y with PDM - VAS ≥ 4 - Normal menstrual cycles 28 ± 7d in recent 3 m	- With SDM or serious diseases - Being pregnant or during lactation - Receiving any other treatment for PDM in recent 2wk - Having a history of delivery	- PDM Consensus Guideline -The revised Chinese National Guideline	Acupuncture (BL54, ST28)	NSAIDs (ibuprofen)	-VAS	3 m / NR	English
12	Yang (2017) (38)	NR, NR	152 (76/76)	- Women aged 18-35y with PDM (pattern of congealing cold with blood stasis) - VAS ≥ 4 -Regular menstrual cycles 28 ± 7d	- Being during lactation, pregnant, or plan to get pregnant - With SDM - Receiving PGSI, drugs and moxibustion in recent 2wk	- Society of Obstetricians and Gynecologists of Canada’s Clinical Guideline of PDM	Moxibustion (CV4, CV8, SP6)	NSAIDs (ibuprofen)	- VAS	3 m / 3 m	English
13	Ding (2021) (39)	NR, 14–35	312 (104/104/104)	- With PDM (pattern of congealing cold with blood stasis) - Aged 14-35y	- Having SDM - Having severe primary diseases - Receiving any treatment in the recent 2wk	NR	Moxibustion (BL23)	NSAIDs (fenbide)	-VAS	4 m / NR	Chinese
14	Wan (2022) (40)	NR, 18–30	60 (30/30)	- Diagnosed with PDM - Single and non-pregnant female aged 18-30y	- With primary diseases, irregular menstrual cycles, contraindications of moxibustion - Receiving any treatment in the recent 1 m	- Diagnosis criteria of PDM in Gynecology in Chinese Medicine - PDM diagnostic criteria by Obstetrics and Gynecology	Moxibustion (GV14, GV2)	NSAIDs (ibuprofen)	-VAS	3 m / NR	Chinese
15	Liu (2019) (41)	NR, 19–27	64 (32/32)	- Diagnosed with PDM - VAS > 4	- Being pregnant or lactating - With SDM or serious primary diseases - Receiving any treatment in the recent 1 m	- Diagnosis criteria of PDM in Gynecology in Chinese Medicine	Warn needling (BL32)	Acupuncture (CV4、SP6, SP8, EX-B8、ST28)	-VAS	3 m/ NR	Chinese
16	Liu (2018) (42)	NR, 18–30	86 (13/13)	- Non-pregnant female aged 18-30y -With a disease duration 0.5-5y -With regular menstrual cycles	- With SDM - Having severe primary diseases - Receiving analgesics 24 h prior to treatment	- Society of Obstetricians and Gynecologists of Canada’s Clinical Guideline of PDM	Moxibustion (lumbosacral and lower abdomen)	NSAIDs (Meloxicam)	-VAS	3 m / NR	Chinese
17	Ye (2022) (43)	NR, NR	74 (37/37)	- With PDM (pattern of congealing cold with blood stasis) - No treatment in the past 30d - Aged 15-35y - Regular menstrual cycles	- Being pregnant or during lactation - Having injuries or infection in the skin, or psychiatric disorders	- Chinese Obstetrics and Gynecology (3^rd^ edition) - Obstetrics and Gynecology (8^th^ edition) - Gynecology in Chinese Medicine (10^th^ edition)	Moxibustion (CV4, CV3, EX-CA1)	NSAIDs (ibuprofen)	- VAS	3 m / 3 m	Chinese
18	Wu (2009) (44)	NR, 14–30	66 (33/33)	- With PDM (pattern of congealing cold with blood stasis) - women aged 14-30y	- With SDM - Lactating women with physical or psychiatric disorders - Receiving any related treatment	- Obstetrics and Gynecology (7^th^ edition) - Clinical Research Guidance for New Drug of Chinese Medicine (1994) - Gynecology in Chinese Medicine (2^nd^ edition)	Moxibustion (CV4, EX-CA1)	Acupuncture (EX-CA1, CV4)	- VAS	3 m / 3 m	Chinese
19	Tang (2012) (45)	NR, 20–23	60 (30/30)	- With PDM (pattern of congealing cold with blood stasis) - Aged 15-30y - VAS ≥ 4 -Regular menstrual cycles 28 ± 7d	- Being pregnant or during lactation - With primary diseases or chronic infectious diseases - Having received any analgesics or sedatives in the recent 2wks	- Clinical Research Guidance on New Drug of Chinese Medicine for DM - Obstetrics and Gynecology (7^th^ edition) - Gynecology in Chinese Medicine	*Tuina* (Spine pinching)	NSAIDs (ibuprofen)	- VAS	3 m / 1 m	Chinese
20	Chang (2020) (46)	NR, NR	90 (45/45)	- With PDM (pattern of congealing cold with blood stasis) - aged 20-45y	- Being pregnant or during lactation - With primary diseases, infectious diseases, or malignant tumor - Having received any treatment in the recent 3 m	- Accurate Diagnosis and Coding of Obstetrics and Gynecology Disease - Exploration and Analysis of TCM Differentiation and Treatment of PDM	Acupuncture (CV6, CV4, SP6, ST29)	NSAIDs (ibuprofen)	- VAS	3 m / NR	Chinese
21	Zhang (2017) (47)	NR, 12–39	70 (35/35)	- With PDM	- Being pregnant or during lactation - With SDM, psychiatric disorders, injuries, or infection on the sites for needling - Having taken analgesics treatment	- Obstetrics and Gynecology - Diagnostic efficacy of standard TCM Syndrome	Electroacupuncture (LI4, SP10, ST36, CV3, BL26, SP8)	NSAIDs (ibuprofen)	- VAS	3 m / 6 m	Chinese
22	Zhang (2020) (48)	NR, 18–30	100 (50/50)	- Non-pregnant female aged 18-30y with PDM -VAS ≥ 4 - With regular menstrual cycles	- With SDM or severe primary diseases - Receiving contraceptives in recent 1y - Receiving drugs or supplements for fatigue during treatment	- Obstetrics and Gynecology (6^th^ edition)	Moxibustion (CV4, SP6)	NSAIDs (ibuprofen)	- VAS	3 m / NR	Chinese
23	Zhang (2019) (49)	NR, 18–28	60 (30/30)	- With PDM (pattern of congealing cold with blood stasis) - With regular menstrual cycles 28 ± 7d - Not receiving analgesics, sedatives or hormonal drugs 2wks prior to treatment	- Being pregnant or during lactation - With SDM, severe primary diseases, or contraindications of moxibustion - Having received contraceptives, intra-uterine device, or planning to become pregnant within 6 m	- Clinical Research Guidance on New Drug of Chinese Medicine for DM - Obstetrics and Gynecology	Moxibustion (CV3, ST36, SP10, SP6, SP8)	NSAIDs (ibuprofen)	-VAS	3 m / NR	Chinese
24	Zhang (2020) (50)	23.16, 16–35	60 (30/30)	- Female aged 12-35y with PDM (pattern of congealing cold with blood stasis) - With regular menstrual period lasting 3-7d with interval of 21-35d - Not received any treatment for PDM in the recent 3 m	- Being pregnant or during lactation - With severe diseases or psychiatric disorders - Having received contraceptives or intra-uterine device - Being allergic to ginger	- Obstetrics and Gynecology (9^th^ edition) - Diagnostic efficacy of standard TCM Syndrome (2017) - Gynecology in Chinese Medicine	Moxibustion (BL31, BL32, BL 33, BL34)	Acupuncture (CV3, SP6, SP8, BL32, EX-B8, CV4, ST29)	- VAS	3 m / 3 m	Chinese
25	Peng (2012) (51)	NR, 18–30	60 (30/30)	- Single female aged 18-35y with PDM	- With severe diseases, contraindications of ibuprofen/acupuncture	- Diagnostic efficacy of standard TCM Syndrome	Acupuncture (SP8, KI5)	NSAIDs (ibuprofen)	- VAS	3 m / NR	Chinese
26	Cao (2011) (52)	NR, 15–28	60 (30/30)	- Non-pregnant female aged 15-35y with PDM - VAS ≥ 4 - With a 6-12 m disease duration -Regular menstrual cycles 28 ± 7 days	- With SDM, severe primary diseases, or contraindications of acupuncture - Having received any treatment for PDM within 1 m	- Society of Obstetricians and Gynecologists of Canada’s Clinical Guideline of PDM - Clinical Research Guidance for New Drug of Chinese Medicine - Gynecology in Chinese Medicine	Acupuncture (EX-B8, BL32, SP8)	NSAIDs (ibuprofen)	- VAS	3 m / 3 m	Chinese
27	Cao (2014) (53)	NR, 16–27	62 (31/31)	- With PDM for more than 3 consecutive menstrual cycles - Aged 16-35y - With regular menstrual cycles 28 ± 7 days	- With SDM or severe diseases - Medical staffs participating in this study - having received any treatment in the recent 15d	- Clinical Research of TCM Gynecology - Therapy of Acupuncture and Moxibustion Course - Obstetrics and Gynecology - Chinese Obstetrics and Gynecology	Acupuncture (BL54, ST28)	NSAIDs (ibuprofen)	- VAS	3 m / NR	Chinese
28	Zhu (2020) (54)	NR, NR	60 (27/33)	- VAS ≥ 6 - Non-pregnant female aged 18-35y	-With severe diseases or SDM - With DM due to sacroiliac joint dislocation -Receive any treatment for DM in the recent 1 m	NR	*Tuina* (rubbing, kneading, trembling, pushing, grasping)	Acupuncture (CV3, SP8, ST29, BL32, CV4, EX-B8, SP6)	- VAS	3 m / NR	Chinese
29	Zhu (2015) (55)	NR, NR	66 (33/33)	- Non-pregnant female aged 16-28y with PDM - With regular menstrual cycles 28 ± 7d	- With SDM - During lactation - With severe diseases - Having received analgesics or hormonal drugs in the recent 2 wks	- Obstetrics and Gynecology (6^th^ edition) - Clinical Research Guidance on New Drug of Chinese Medicine for DM (1993)	Acupuncture (CV4, CV6, CV12, CV10, KI13, EX-CA1)	NSAIDs (ibuprofen)	-VAS	3 m / 1 m	Chinese
30	Li (2014) (56)	NR, NR	20 (10/10)	- Nonpregnant female aged 19-30y with PDM - VAS ≥ 4 / CMSS ≥10 - With regular menstrual cycles 28 ± 7d and feel pain within 48 h of menstruation	- With other diseases - With previous experience of needling - Having received any analgesics in 1wk prior to treatment	- Guidelines for Clinical Research on New Chinese Medicines for DM - Obstetrics and Gynecology	Acupuncture (SP6)	Sham acupuncture (SP6)	-VAS	8 min / NR	Chinese
31	Li (2017) (57)	19, 18–20	70 (35/35)	- Women aged18-45y with PDM - regular menstrual cycles 28 ± 7 days - Not received any other treatment	- With SDM, severe physical, or psychiatric disorders - Being pregnant or during lactation	- Criteria for Diagnosis and Treatment of TCM Syndrome - Obstetrics and Gynecology	Auricular therapy (TF2, CO18, TF4, CO12)	NSAIDs (ibuprofen)	- VAS	3 m / NR	Chinese
32	Lin (2019) (58)	NR, NR	62 (30/32)	- Women aged18-30y - With PDM (pattern of congealing cold with blood stasis) - With regular menstrual cycles 28 ± 7d	- With severe primary diseases or psychiatric disorders - Having received any treatment for PDM in the recent 1 m	- Society of Obstetricians and Gynecologists of Canada’s Clinical Guideline of PDM - Diagnostic and Curative Criteria for Gynecological Diseases in TCM	Acupuncture (SP6, CV4, GV4, CV3, SP6, BL23)	NSAIDs (ibuprofen)	- VAS	3 m / 3 m	Chinese
33	Lin (2020) (59)	NR, NR	110 (55/55)	- Women aged15-35y with PDM -Regular menstrual cycles 28 ± 7 days	- Being pregnant or during lactation - With SDM, severe primary diseases, or psychiatric disorders - Having receive any treatment in the recent 1 m	- Obstetrics and Gynecology - Gynecology in Chinese Medicine	Warm needling (EX-CA1, CV4, ST36, SP6)	NSAIDs (ibuprofen)	- NRS	3 m / NR	Chinese
34	Lin (2020) (60)	NR, 19–32	66 (33/33)	- Nonpregnant female aged 18-35y with PDM - Regular menstrual cycles 28 ± 7 days	- With primary diseases or infection - Having received contraceptives or intra-uterine device - Once received endocrine-related medication or injection	- Clinical Research Guidance for New Drug of Chinese Medicine - Obstetrics and Gynecology (8^th^ edition) - Gynecology in Chinese Medicine	Warm needling (EX-B8, GV14, BL17)	NSAIDs (fenbide)	- VAS	3 m / 3 m	Chinese
35	Liang (2021) (61)	NR, NR	60 (30/30)	- Nonpregnant female aged 16-32y with PDM -VAS ≥ 4 - Menarche over 2y with regular menstrual cycles 28 ± 7 days - Abdominal pain in the period	- With contraindications of ibuprofen - With severe primary diseases and hypertension - Receive any treatment for DM	- Obstetrics and Gynecology (8^th^ edition) - Gynecology in Chinese Medicine (9^th^ edition) - Clinical Research Guidance for New Drug of Chinese Medicine	Moxibustion (CV8)	NSAIDs (ibuprofen)	- VAS	3 m / 3 m	Chinese
36	Fan (2014) (62)	NR, 18–35	60 (30/30)	- Women aged 18-35y with PDM (pattern of congealing cold with blood stasis) - VAS ≥ 4 - Regular menstrual cycles 28 ± 7 d	- Being pregnant or during lactation - With SDM, severe primary diseases, psychiatric disorders, or contraindications of moxibustion - Received drugs for DM in the recent 2wks	- Clinical Research Guidance for New Drug of Chinese Medicine - Diagnostic efficacy of standard TCM Syndrome	Moxibustion (CV4, CA7)	Acupuncture (CV4, CA7)	- VAS	3 m / 3 m	Chinese
37	Tang (2015) (63)	NR, 18–27	60 (30/30)	- Regular menstrual cycles 28 ± 7 days with PDM	- With severe primary diseases or psychiatric disorders - Taking analgesics before or during treatment	- Clinical Research Guidance for New Drug of Chinese Medicine - Obstetrics and Gynecology	Acupuncture (SP6, SP8, CV4, LI4)	NSAIDs (ibuprofen)	- VAS	3–6 m / NR	Chinese
38	Wen (2021) (64)	NR, 17–34	60 (30/30)	- Women aged16-35y - With PDM (pattern of congealing cold/ qi stagnation with blood stasis) -regular menstrual cycles 28 ± 7 d	-With SDM, bleeding tendencies, or coagulation disorders - Having received any treatment for PDM in the recent 3 m	- Gynecology in Chinese Medicine - Diagnosis of Obstetrics and Gynecology Diseases	Acupuncture (LR3, BL18, LR6, CV4)	NSAIDs (ibuprofen)	-VAS	3 m / 3 m	Chinese
39	Wang (2019) (65)	25.26, 15–35	60 (30/30)	- Women aged18-35y with PDM - Not received any treatment in the recent 1 m	- Being pregnant or lactation - With irregular menstrual cycles - With severe primary disease - Having received analgesics or hormonal drugs	- Obstetrics and Gynecology (8^th^ edition) - Criteria for Diagnosis and Treatment of TCM Syndrome	Acupuncture (CV12, CV10, CV6, CV4, ST24, ST26, GB31)	NSAIDs (ibuprofen)	- VAS	3 m / 3 m	Chinese
40	Wang (2018) (66)	NR, NR	120 (60/60)	- Women aged 15-28y - VAS ≥ 4 - With regular menstrual cycles - Not received medicine in the recent 1wk	- Being pregnant or during lactation - With SDM, severe primary diseases, psychiatric disorders, pelvic inflammation, or organic disorders - Using other intervention for DM	- Obstetrics and Gynecology - Guidelines for Clinical Research on New Chinese Medicines for DM - Diagnostic efficacy of standard TCM Syndrome	Moxibustion (CV4, SP6, BL32)	NSAIDs (ibuprofen)	- VAS	3 m / NR	Chinese
41	Bai (2018) (67)	NR, 16–33	80 (40/40)	- With PDM (pattern of congealing cold with blood stasis) -VAS ≥ 4 - Regular menstrual cycles 28 ± 7 d	- Being pregnant or during lactation -With SDM -With serious medical diseases	- Obstetrics and Gynecology - Therapy of Acupuncture and Moxibustion Course	Moxibustion (BL31, BL32, BL33, BL34)	NSAIDs (ibuprofen)	- VAS	3 m / NR	Chinese
42	Sheng (2019) (68)	NR, NR	72 (36/36)	- Women aged 16-35y with PDM - With regular menstrual period of 3-7d with an interval of 21-35d - Not receive analgesics, sedatives or hormonal drugs prior to treatment	- Being pregnant or during lactation - With SDM, severe primary diseases, or contraindications of acupuncture - Receiving PDM medicine or intra-uterine devices - Having received oral prostaglandin synthase inhibitors within 2wks	- Evidence-based Clinical Practice Guideline of Acupuncture and Moxibustion for PDM - Gynecology in Chinese Medicine - Criteria for Diagnosis and Treatment of TCM Symptom	Acupuncture (SP8, EX-B8, SP6, BL32, EPC6)	NSAIDs (ibuprofen)	- VAS	3 m / 3 m	Chinese
43	Shi (2022) (69)	NR, 19–28	60 (30/30)	- With PDM of cold damp pattern - Nonpregnant female aged 16-28y - With regular menstrual cycles 28 ± 7	- With SDM, serious diseases of vital organs, blood and circulatory systems, or psychiatric disorders - Having received analgesics in the recent 2 m	- Obstetrics and Gynecology (9^th^ edition) - Clinical Research Guidance for New Drug of Chinese Medicine	Scraping therapy (GV4, EX-B8, CV6, CV2)	NSAIDs (ibuprofen)	- NRS	3 m / NR	Chinese
44	Shi (2017) (70)	NR, NR	44 (22/22)	- With PDM (pattern of congealing cold with blood stasis) - Nonpregnant female aged 18-35y - VAS = 5–9 - With regular menstrual cycles 21-35d - Disease duration ≥3 m	- With SDM, prolonged illness, weakness, emaciation, or severe diseases - Injuries or infection on the sites for acupuncture - Having received any treatment in the recent 1 m	- Obstetrics and Gynecology (8^th^ edition) - Gynecology in Chinese Medicine	Acupuncture (CV6, CV4, SP6)	NSAIDs (ibuprofen)	- VAS	3 m / NR	Chinese
45	Lou (2020) (71)	NR, NR	60 (30/30)	- With PDM (pattern of congealing cold with blood stasis) - Single and nonpregnant female aged 16-35y - With regular menstrual cycles 28 ± 7d	- With SDM, organic disorders, or psychiatric disorders - With contraindications to ibuprofen or acupuncture - Having received any treatment in the recent 1 m	- Obstetrics and Gynecology (7^th^ edition) - Diagnostic efficacy of standard TCM Syndrome - Gynecology in Chinese Medicine (2nd edition)	*Acupressure* (FV, CV, BV, CV6, EX-CA1, SP10, ST34, ST36, SP6)	NSAIDs (ibuprofen)	- VAS	3 m / NR	Chinese
46	Zhai (2020) (72)	NR, 18–30	78 (39/39)	- Nonpregnant female aged 18-30y with PDM - VAS > 2 - With regular menstrual cycles 28 ± 7d - Not received any treatment for PDM	- Being pregnant, in lactation - once experienced miscarriage and stillbirth - With severe primary diseases or psychiatric disorders -Having taken analgesics in the recent 24 h	NR	Acupressure (carpus-ankle)	Sham acupressure	- VAS	30 min / NR	Chinese
47	Xiao (2016) (73)	NR, 12–30	60 (30/30)	- With PDM (pattern of congealing cold/ qi stagnation with blood stasis) -Nonpregnant female aged 12-30y	- With SDM, severe primary diseases, or psychiatric disorders - having received contraceptives, intra-uterine device, or planning to become pregnant	- Gynecology in Chinese Medicine - Obstetrics and Gynecology	Acupuncture (ST25, ST26, ST27, ST28, CV4, CV3)	NSAIDs (ibuprofen)	-VAS	3 m / 3 m	Chinese
48	Fan (2021) (74)	NR, 18–35	60 (30/30)	- nonpregnant or nonlactating women aged 18-35y with PDM (pattern of congealing cold/ qi stagnation with blood stasis) - with regular menstrual cycles 28 ± 7d	- with severe primary diseases or contraindications of moxibustion - having received contraceptives or intra-uterine device - having received any treatment in the recent 1 m	- Society of Obstetricians and Gynecologists of Canada’s Clinical Guideline of PDM - Gynecology in Chinese Medicine	Moxibustion (CV6, CV4, ST36)	NSAIDs (ibuprofen)	- VAS	3 m / 3 m	Chinese
49	Jia (2017) (75)	NR, NR	120 (60/60)	- female aged 16-30y with PDM - VAS > 3 - With regular menstrual period of 3-7d and an interval of 21-35d - Not received analgesics, sedatives or hormonal drugs in the recent 1wk	- Being pregnant or during lactation - With SDM, severe primary diseases, psychiatric disorders, or contraindications of acupuncture - Using intra-uterine device and medicine for DM in the recent 1 m	- Evidence-based Clinical Practice Guideline of Acupuncture and Moxibustion for PDM - Gynecology in Chinese Medicine (7^th^ edition)	Acupuncture (SP8, EX-B8, SP6, BL32, PC6)	NSAIDs (ibuprofen)	- VAS	6 m / NR	Chinese
50	Hao (2018) (76)	NR, 16–35	60 (30/30)	- Female aged 15-35y with PDM - VAS ≥ 3 -With regular menstrual cycles 28 ± 7d	- Being pregnant or during lactation - With SDM, severe primary diseases, psychiatric disorders, or contraindications of acupuncture	- Obstetrics and Gynecology - Gynecology in Chinese Medicine	Acupuncture (SP6, ST36, LI4, PC6, RN8)	NSAIDs (ibuprofen)	-VAS	3 m / NR	Chinese
51	Guo (2021) (77)	NR, NR	82 (41/41)	- With PDM (pattern of congealing cold with blood stasis) - Aged 15-35y - VAS ≥ 6	- Being pregnant or during lactation - With SDM, severe diseases, and contraindications to moxibustion - Having received treatment for DM in the recent 2 m	- Obstetrics and Gynecology - Gynecology in Chinese Medicine	Moxibustion (CV8, CV2, CV4, CV6, ST36)	NSAIDs (ibuprofen)	- VAS	3 m / 1 m	Chinese
52	Zhong (2017) (78)	NR, NR	64 (33/31)	- Female with PDM (pattern of congealing cold with blood stasis) - Menarche over 1y	- With severe primary diseases or psychiatric disorders	NR	Warm needling (CV4, ST29, SP6)	NSAIDs (Ibuprofen)	- VAS	3 m / NR	Chinese
53	Chen (2011) (79)	NR, NR	60 (30/30)	- Single and nonpregnant female aged 15-32y with PDM 6 m-15y - VAS ≥ 4 - with regular menstrual cycles 28 ± 7d	- With SDM, severe primary diseases, or organic disorders - with contraindication of aspirin - having received any analgesics in the recent 24 h	- Society of Obstetricians and Gynecologists of Canada’s Clinical Guideline of PDM - Diagnostic efficacy of standard TCM Syndrome	Acupressure (CV6, CV4, ST25, SP8)	NSAIDs (ibuprofen)	- VAS	3 m / NR	Chinese
54	Chen (2014) (80)	NR, NR	80 (40/40)	- With PDM - Not receive analgesics or hormonal drugs 3 m prior to treatment	- With SDM, severe primary diseases, or drugs allergy	-Diagnostic efficacy of standard TCM Syndrome	Acupuncture (SP8, CV3, SP6, KI3)	NSAIDs (ibuprofen)	-VAS	3 m / NR	Chinese
55	Chen (2022) (81)	NR, 20–28	42 (21/21)	- Nonpregnant female aged 18-30y with PDM - VAS ≥ 4 - with regular menstrual cycles 28 ± 7d	- With SDM, severe primary diseases, or contraindications to magnetic resonance imaging - Having taken medicine1m prior to treatment	- Society of Obstetricians and Gynecologists of Canada’s No.345 PDM Consensus Guideline	Acupuncture (SP6, CV4)	Waitlist	- VAS	3 m / NR	Chinese
56	Han (2015) (82)	NR, NR	60 (30/30)	- Nonpregnant female aged 19-24y with PDM - VAS ≥ 4 - CMSS ≥1 - with regular menstrual cycles 28 ± 7d	- Being pregnant or during lactation - With severe primary diseases or psychiatric disorders - Medical staff participating in this study - Having received acupuncture or analgesics 1wk prior to treatment	- Clinical Research Guidance for New Drug of Chinese Medicine - Obstetrics and Gynecology	Acupuncture (SP6)	Sham acupuncture (SP6)	- VAS	3 m / NR	Chinese
57	Wei (2019) (83)	NR, NR	86 (43/43)	- Nonpregnant female aged 18-30y with PDM - VAS ≥ 4 - disease duration ≥3 m	- With SDM, irregular menstrual cycles, severe primary diseases, or contraindications of ibuprofen/acupuncture	- Obstetrics and Gynecology - Clinical Research Guidance for New Drug of Chinese Medicine	Acupuncture (BL31, BL32, BL33, BL34)	NSAIDs (ibuprofen)	- VAS	3 m / 3 m	Chinese

I, intervention group; C, control group; NSAID, nonsteroidal anti-inflammatory drug; VAS, visual analogue scale; NR, not reported; PDM, primary dysmenorrhea: SDM, secondary dysmenorrhea; DM, dysmenorrhea; VMSS, verbal multidimensional scoring; COC, combined oral contraceptive pill; PGSI, prostaglandin synthetase inhibitor; CMSS, Cox menstrual symptom scale; MPQ, McGill pain questionnaire; BPI, brief pain inventory; TCM, traditional Chinese medicine.

### 3.3 Risk of bias

**Figure 2:**
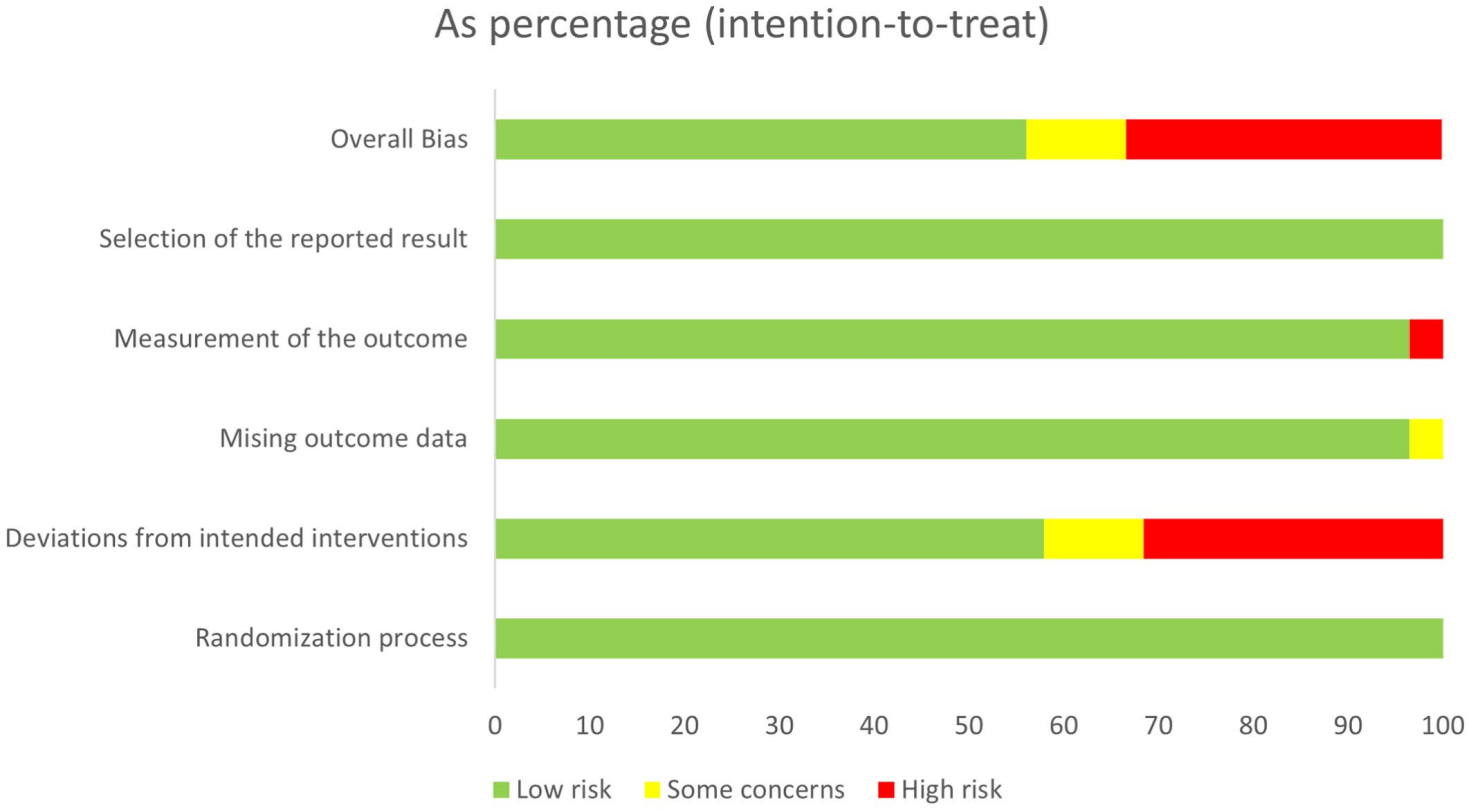
Summary of risk of bias (RoB) of the included studies.

Figure 2 presents a summary of the methodological quality of the included studies. Thirty-two RCTs were rated as low risk of bias (28, 31, 33, 34, 36, 39–43, 46, 47, 49, 51, 56, 59, 61–66). Nineteen studies were rated as high risk of bias due to the application of inappropriate data analysis methods (27, 29, 30, 32, 45, 48, 50, 54, 55, 57, 58, 60, 68, 71, 72, 81, 83) or unblinding of the outcome assessors (35, 81). The remaining six studies were rated as having certain concerns because of the insufficient information on data analysis methods (37, 38, 44, 52, 53, 77) or the high dropout rate (57, 75).

### 3.4 Network diagram

**Figure 3:**
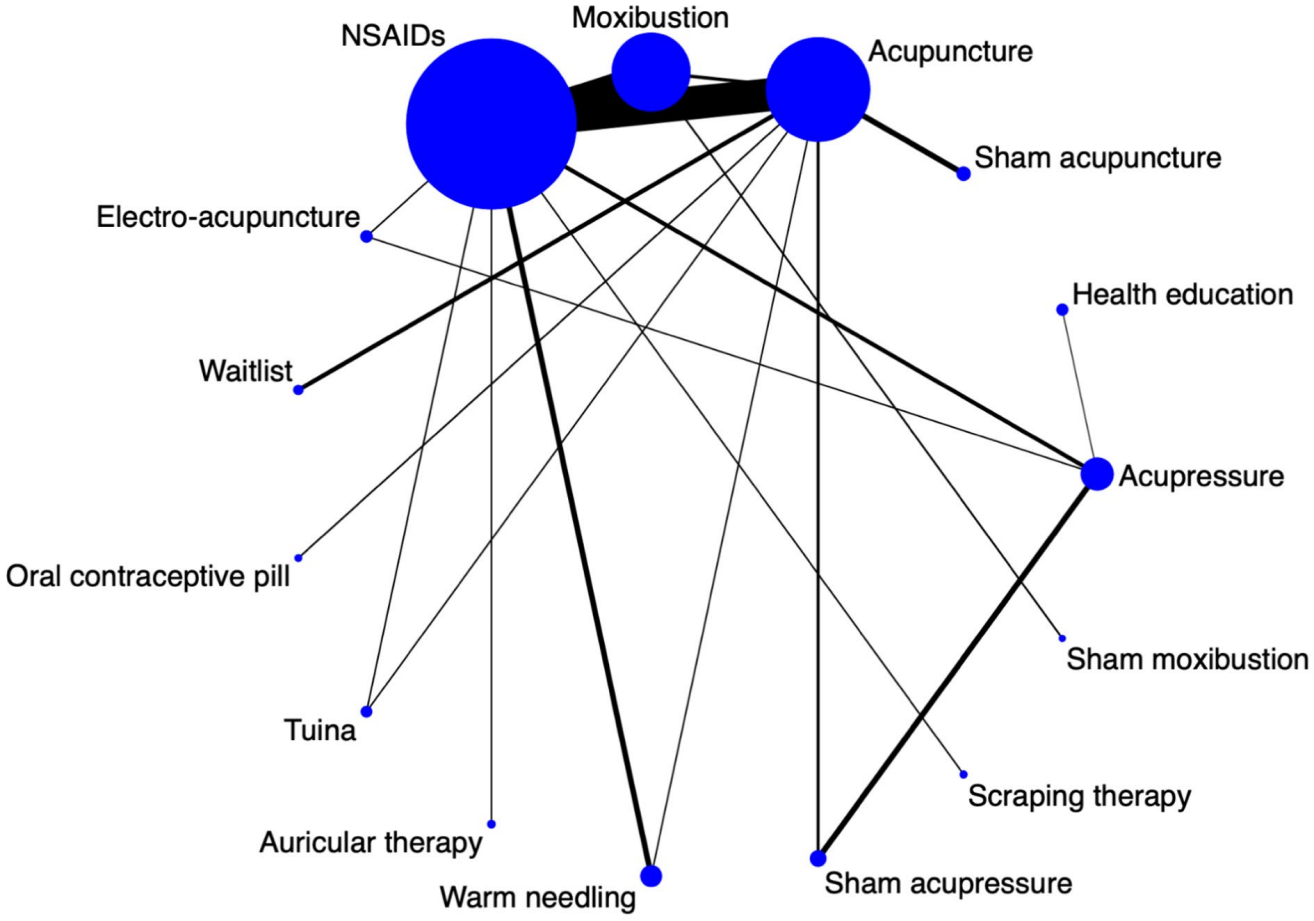
A network diagram for all interventions.

A network diagram was drawn based on 15 interventions, including 3,903 participants. A total of 105 pairs of comparisons were formed (including 18 pairs of direct comparisons and 87 pairs of indirect comparisons). Nineteen RCTs reported a comparison between acupuncture and NSAIDs, and 12 RCTs reported the comparison between moxibustion and NSAIDs. Direct comparisons between 9 pairs of comparisons occurred only once. Among these interventions, NSAIDs had the largest sample size (*n* = 1,426), followed by acupuncture (*n* = 865), moxibustion (*n* = 651), and warm needling (*n* = 151). The data are shown in Figure 3. (Supplementary Appendix 3).

### 3.5 Heterogeneity and inconsistency test

Conceptual heterogeneity and inconsistency were low across the network given that all the included studies had the same study design, participants, and outcomes. Statistical heterogeneity was high in several pairwise comparisons (Supplementary Appendix 4). Supplementary Appendix 5 shows the detailed information on the local inconsistency test. A node-splitting test was conducted for local inconsistency analysis, and 19 pairs of mixed comparisons (including direct and indirect) were analyzed. The results showed no statistical inconsistency in each pair of direct comparison. Sensitivity analysis indicated that the findings were robust (Supplementary Appendix 6).

### 3.6 NMA

**Figure 4:**
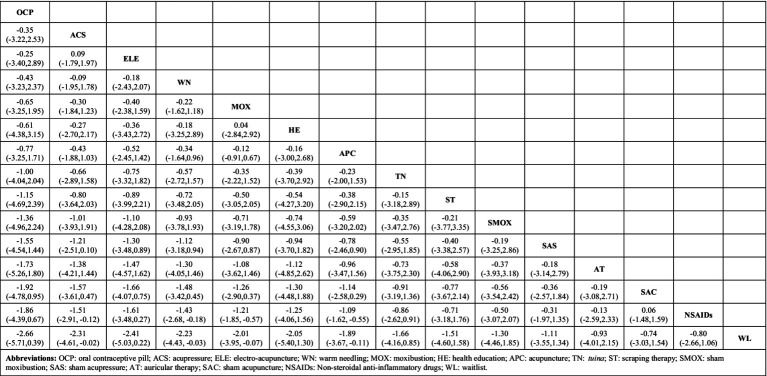
The network meta-analysis for all interventions.

As shown in Figure 4, the estimated effect of the NMA for each intervention on relieving PD pain was generated. Compared with NSAIDs and waitlist, significantly better effects were found in acupressure [SMD = −1.51, 95% CI (−2.91, −0.12)/SMD = −2.31, 95% CI (−4.61, −0.02)], warm needling [SMD = −1.43, 95% CI (−2.68, −0.18)/SMD = −2.23, 95% CI (−4.43, −0.03)], moxibustion [SMD = −1.21, 95% CI (−1.85, −0.57)/SMD = −2.10, 95% CI (−3.95, −0.07)], and acupuncture [SMD = −1.09, 95% CI (−1.62, −0.55)/SMD = −1.89, 95% CI (−3.67, −0.11)].

### 3.7 Probability ranking

**Figure 5:**
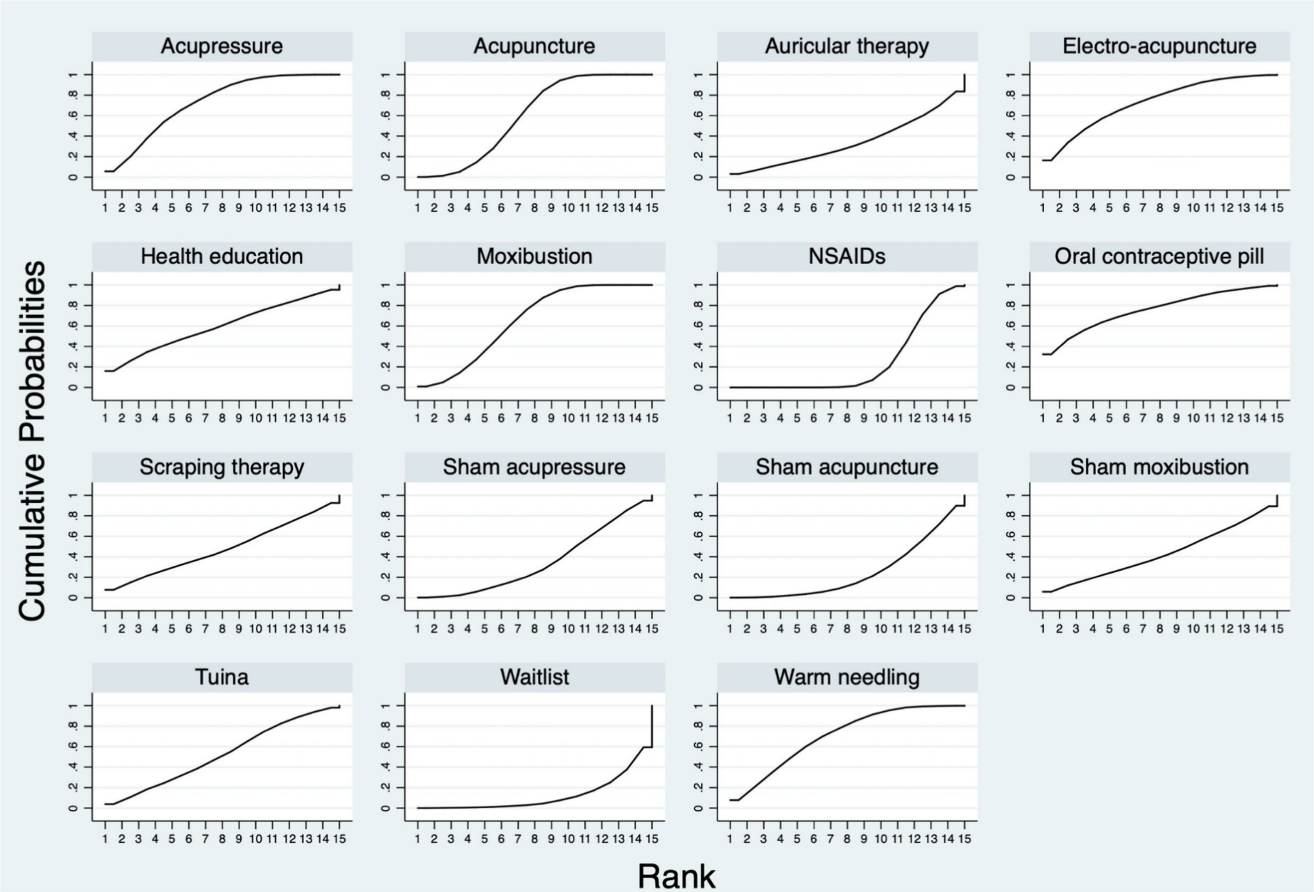
The probability ranking for all the interventions.

Figure 5 shows the SUCRA probability ranking for the different treatment effects of all interventions. For the 15 interventions, the SUCRA value predicted the possibility of different interventions as the best treatment on the management of PD pain, and the ranking was as follows: oral contraceptive pill (75.8%), electro-acupuncture (73.0%), acupressure (72.9%), warm needling (70.6%), moxibustion (64.9%), acupuncture (60.0%), health education (59.7%), *tuina* (52.3%), scarping therapy (48.0%), sham moxibustion (43.1%), sham acupressure (34.8%), auricular therapy (34.1%), sham acupuncture (24.9%), NSAIDs (23.9%), and waitlist (12.1%).

### 3.8 Safety

Information on the safety of different interventions was collected and assessed based on adverse events reported in RCTs. Nineteen RCTs reported adverse events on acupuncture, warm needling, NSAIDs, and sham acupressure. For acupuncture and warm needling, adverse events included local bleeding or hematoncus (8 RCTs) (36, 37, 51, 53, 58, 60, 68, 73), headache or myalgia (1 RCT) (36), fever (1 RCT) (36), nausea (1 RCT) (46), and nervousness in the first treatment session (1 RCT) (71). Adverse events of NSAIDs incorporated gastrointestinal symptoms (13 RCTs) (36, 37, 46, 48, 51–53, 65, 68, 69, 73, 75, 83), vaginal bleeding (1 RCT) (36), weight gain (1 RCT) (36), breast engorgement (1 RCT) (36), hypermenorrhea (1 RCT) (48), rash (1 RCT) (52), and dizziness and tinnitus (3 RCTs) (68, 73, 83). An RCT also reported that the analgesic effect of medication faded away with repetitive use in some cases (31). An RCT mentioned that 3 participants dropped out from the sham acupressure control group due to severe pain (30, 72), whereas another RCT reported that 2 participants dropped out from the sham acupressure group without providing the specific reasons (30). Details on the adverse events of the interventions are in Supplementary Appendix 7.

### 3.9 Certainty of evidence

The evidence quality of all 18 comparisons was evaluated by GRADEpro. High-quality evidence was found in two comparisons on PD pain symptoms, namely, acupuncture versus placebo and electroacupuncture versus NSAIDs. The evidence quality was evaluated as moderate in six comparisons: acupressure versus placebo, moxibustion versus NSAIDs, moxibustion versus acupuncture, *tunia* versus acupuncture, warm needling versus NSAIDs, and scraping therapy versus NSAIDs. The evidence quality of the rest of the 10 comparisons was evaluated as low or very low. The most common reason for downgrading the evidence quality was imprecision, followed by high heterogeneity and high risk of bias. Details of the SoF table are in Supplementary Appendix 8.

### 3.10 Publication bias

The publication bias is shown in Appendix 9. The results showed that the studies were roughly symmetrical around the center line. Egger’s test (*p* = 0.162) showed no significant risk of publication bias as well.

## 4 Discussion

Numerous clinical trials and systematic reviews have investigated the effects of meridian-based TCM interventions for PD pain and suggested that these interventions can reduce PD pain intensity, duration, and related symptoms, with relatively few adverse events. However, this work is the first study to quantitatively summarize and compare the effects of the meridian-based TCM interventions via NMA. In PD pain management, the effects of acupressure, acupuncture, and moxibustion were superior to those of NSAIDs and waitlist; acupressure was more effective than placebo; and warm needling was superior to both NSAIDs and waitlist.

Previous reviews have evaluated the effects of meridian-based TCM interventions for PD pain and found similar results to this study. A systematic review of acupuncture-related therapies (acupuncture, acupoint injection, acupressure, and moxibustion) for PD indicated that acupuncture was significantly more effective than placebo (WMD = −0.57, 95% CI [−0.76, −0.38]) and NSAIDs (WMD = −0.19, 95% CI [−0.37, −0.01]) in PD pain management, and acupressure was more effective than placebo (WMD = −0.91, 95% CI [−1.78, −0.04]). Nevertheless, the reliability of their review may be compromised because it included a combination of randomized and non-randomized trials with fewer interventions, trials, and comparisons. Moreover, most of the studies included were deemed low in methodology (84). Another systematic review on the effects and safety of acupuncture and moxibustion for PD demonstrated that acupuncture (MD = −1.93; 95% CI [−2.80, −1.06]) and moxibustion (MD = −2.67; 95% CI [−4.96, −0.38]) are more effective in managing PD pain than the control via VAS; however, the review combined all control interventions as one for their meta-analysis (85). Similarly, Jiang et al. conducted a systematic review of RCTs on acupressure for PD and reported that acupressure is better at improving pain with VAS compared with placebo or waitlist (MD: −1.41, 95% CI [−1.61, −1.21]), but the study found no difference in pain relief between acupressure and NSAIDs. This discrepancy may be due to the small number of RCTs with modest sample included, which hindered the detection of treatment effects in their systematic review (86). The present study’s findings suggested that electroacupuncture may be an optimal intervention strategy for PD pain; however, direct comparisons among interventions were few based on a limited number of studies.

For safety issues, the current study provided evidence that meridian-based TCM interventions are safe with no severe adverse events for the treatment of PD, and the reported adverse events focused on acupuncture and warm needling. These results were in accordance with previous studies (33, 84, 85). Fainting, hematoma or bleeding, sticking of needle, and needling sensation after acupuncture are common situations during acupuncture treatment in clinics or research (87). These situations might be related to irregular acupressure practice of practitioners, participants’ mental and physical conditions, and participant–practitioner interactions (88). All the abovementioned adverse events are mild and can be handled well under appropriate management. A systematic review examining the adverse events of meridian-based TCM interventions between 2000 and 2011 (117 studies, 25 countries, and 294 cases with adverse events in acupuncture) emphasized the significance of acupuncture practice guidelines (89). Therefore, to minimize adverse events, practitioners should strictly follow the operation guidelines, observe the situation of patients, and communicate with them actively during acupuncture treatment. Standard reporting form can be adopted in future studies to systematically document the occurrence and severity of AE (90).

The good statistical consistency observed in the NMA suggested that the variation in the treatment effects of the meridian-based TCM interventions is predictable and not due to chance (91). However, the statistical heterogeneity in the pairwise comparisons within the NMA is high. Several appropriate statistical methods were applied to identify the sources of heterogeneity. Sensitivity analysis indicated the robustness of the overall results of the meta-analysis (92). Publication bias was suggested from the funnel plot and Egger’s test. Considering that the overall methodological quality of the included studies was high, the high statistical heterogeneity observed in the pairwise comparisons could be attributed to the variability in sample size or the different durations of intervention among various studies (93). The heterogeneity may also arise from differences in demographic characteristic of participants with PD among the study populations (94). Future systematic reviews may limit the age range of the participants, define the optimal information size (for example, 400 as suggested by GRADEpro handbook) (95), and set the treatment duration and frequency. Special attention should be paid to the clinical trials with a large effect size but a small sample size (96, 97).

For the quality of methodology of the included studies, factors that led to high risk of bias included the application of inappropriate data analysis methods, unblinding of the outcome assessors, insufficient information on data analysis methods, and high dropout rate. The factors that rated down the evidence quality of this study by GRADEpro contained imprecision, high heterogeneity, and risk of bias. Further RCTs should give particular emphasis on these several domains, applying the intention-to-treat approach for data analysis to eliminate or reduce bias in treatment effects arising from missing data (98), blinging the outcome assessors to reduce detection bias (99), reporting the results completely to avoid selective reporting bias (e.g., selective outcome reporting, selective analysis reporting, and lack of reporting of adverse events and dropout reasons) (100), and making sure that the sample size has the power to demonstrate the smallest effect of intervention (101).

### 4.1 Implication

The findings of this study can implicate PD patients, healthcare providers, researchers, and policy makers. The study provides a potential ranking of the effects of multiple interventions (e.g., meridian-based TCM interventions and conventional western medicine) for relieving PD pain, as well as their adverse events. The information could be a reference for PD patients when choosing interventions. Health care providers may use the findings to inform their clinical decisions and improve patient outcomes. Researchers may find some research gaps from the findings. For example, the results indicated that acupressure, moxibustion, and warm needling may also have significant effects on PD pain, but relevant evidence is very limited. More clinical trials can be conducted to examine their effects on PD pain management. Researchers could further explore the effects of meridian-based TCM interventions on other outcomes in patients with PD. Policy makers in the field of women’s health may focus on the safety and potential effects of meridian-based TCM interventions and highlight the importance of integrating these interventions into clinical practice guidelines and health care policies.

#### 4.2 Strength

The present study has several strengths. First, the study strictly defined the inclusion criteria, and the overall methodological quality of the included studies was high. Second, we limited the outcome as PD pain, providing a clear and specific research question that allowed for a focused assessment of the current evidence. Third, evidence of both effects and adverse events was collected, enabling readers to obtain knowledge of meridian-based TCM interventions on PD pain. Additionally, the review included studies published in both English and Chinese, which increased the generalizability of the findings and avoided potential language bias.

### 4.3 Limitation

Several limitations of this study should be acknowledged. First, as a result of the diversity of intervention types, the number of studies using the same interventions in some direct comparisons was relatively small. Besides, the duration of the interventions varied across the included studies, which may affect the effects of the interventions. These factors may subsequently influence the reliability of the findings. Second, the study only focused on the severity of PD pain as the sole outcome. Evidence on the effects of meridian-related interventions on other associated symptoms such as menstrual blood flow, sleep quality, and mood was not reviewed. Lastly, the statistical heterogeneity observed among different comparisons was high, suggesting the presence of substantial variability in the results and limiting the generalizability of the findings.

## 5 Conclusion

For the management of PD pain, the effects of acupressure, acupuncture, warm needling, and moxibustion were superior to those of NSAIDs and waitlist. Furthermore, based on probability ranking, oral contraceptive pill, electro-acupuncture, acupressure, and warm needling demonstrated higher probabilities of being better interventions. Meridian-based TCM interventions are safe, and no severe related adverse events were detected. More high-quality clinical trials are needed to provide more robust evidence of this NMA.

## Data Availability

The original contributions presented in the study are included in the article/Supplementary material, further inquiries can be directed to the corresponding author/s.
