# Supplementary material for: Traditional Chinese medicine interventions based on meridian theory for pain relief in patients with primary dysmenorrhea: a systematic review and network meta-analysis
Source: Front Med (Lausanne). 2024 Sep 5;11:1453609. doi: 10.3389/fmed.2024.1453609 (PMC11411804; doi:10.3389/fmed.2024.1453609)

Appendix 1. Search strategies

Ovid MEDLINE

(massag* or anmo or acupress* or tuina or acupunct* or electroacupunct* or electro-acupunct* or acupoint* or meridia* or auricular or needl* or moxibustion or moxa).mp. and (dysmenorr* or menstrua* pain or period cramp or period pain* or menstrua* distress or period distress or menstrua* distress).m_titl. [mp=title, book title, abstract, original title, name of substance word, subject heading word, floating sub-heading word, keyword heading word, organism supplementary concept word, protocol supplementary concept word, rare disease supplementary concept word, unique identifier, synonyms, population supplementary concept word, anatomy supplementary concept word]

Records retrieved: 295

Embase

(massag*:ti OR anmo:ti OR acupress*:ti OR tuina:ti OR acupunct*:ti OR electroacupunct*:ti OR 'electro acupunct*':ti OR acupoint*:ti OR meridia*:ti OR auricular:ti OR needl*:ti OR moxibustion:ti OR moxa:ti) AND (dysmenorr*:ti OR 'menstrua* pain':ti OR 'period cramp':ti OR 'period pain*':ti OR 'period distress':ti OR 'menstrua* distress':ti)

Records retrieved: 309

Health Technology Assessment Database (HTA)

((massag* OR anmo OR acupress* OR tuina OR acupunct* OR electroacupunct* OR electro-acupunct* OR acupoint* OR meridia* OR auricular OR needl* OR moxibustion OR moxa))[Title] AND ((dysmenorr* OR menstrua* pain OR period cramp OR period pain* OR menstrua* distress OR period distress OR menstrua* distress))[Title]

Records retrieved: 19

Cochrane Central Register of Controlled Trials

Word variations have been searched.

Search string: (massag* OR anmo OR acupress* OR tuina OR acupunct* OR electroacupunct* OR electro-acupunct* OR acupoint* OR meridia* OR auricular OR needl* OR moxibustion OR moxa) in Title Abstract Keyword AND (dysmenorr* OR menstrua* pain OR period cramp OR period pain* OR menstrua* distress OR period distress OR menstrua* distress) in Title Abstract Keyword

Records retrieved:55

Web of Science

(TI=((massag* OR ando OR acupress* OR tuina OR acupunct* OR electroacupunct* OR electro-acupunct* OR acupoint* OR meridia* OR auricular OR needl* OR moxibustion OR mona))) AND TI=((dysmenorr* OR menstrua* pain OR period cramp OR period pain* OR menstrua* distress OR period distress OR menstrua* distress))

Records retrieved: 186

the China National Knowledge Infrastructure (CNKI)

#1

(篇名: 按摩 + 针刺 + 推拿 + 针灸 + 电针 + 穴位 + 穴位按摩 + 经络 + 耳穴 + 针刺 + 艾灸 + 艾草 (模糊)) AND (篇名: 痛经 + 月经疼痛 + 经期痉挛 + 经期疼痛 + 经期困扰 + 月经困扰 (模糊))

Records retrieved: 2346

#2

(篇名: 按摩 + 针刺 + 推拿 + 针灸 + 电针 + 穴位 + 穴位按摩 + 经络 + 耳穴 + 针刺 + 艾灸 + 艾草 (精确)) AND (篇名: 痛经 + 月经疼痛 + 经期痉挛 + 经期疼痛 + 经期困扰 + 月经困扰 (精确))

Records retrieved: 33

Wanfang Data

#1

题名:(按摩 OR 针刺 OR 推拿 OR 针灸 OR 电针 OR 穴位 OR 穴位按摩 OR 经络 OR 耳穴 OR 针刺 OR 艾灸 OR 艾草) and 题名:(痛经 OR 月经疼痛 OR 经期痉挛 OR 经期疼痛 OR 经期困扰 OR 月经困扰)
Records retrieved: 2507
#2
题名:(按摩 OR 针刺 OR 推拿 OR 针灸 OR 电针 OR 穴位 OR 穴位按摩 OR 经络 OR 耳穴 OR 针刺 OR 艾灸 OR 艾草) and 题名:(痛经 OR 月经疼痛 OR 经期痉挛 OR 经期疼痛 OR 经期困扰 OR 月经困扰) 分类号:" 医药、卫生"
Records retrieved: 2341
#3
题名:(按摩 OR 针刺 OR 推拿 OR 针灸 OR 电针 OR 穴位 OR 穴位按摩 OR 经络 OR 耳穴 OR 针刺 OR 艾灸 OR 艾草) and 题名:(痛经 OR 月经疼痛 OR 经期痉挛 OR 经期疼痛 OR 经期困扰 OR 月经困扰) not 题名:(心得体会 OR 病例 OR 经验撷要 OR 经验撷菁 OR 教学 OR 体会) 分类号:" 医药、卫生"
Records retrieved: 2274

PubMed
((massag*[Title] OR anmo[Title] OR acupress*[Title] OR tuina[Title] OR acupunct*[Title] OR electroacupunct*[Title] OR electro-acupunct*[Title] OR acupoint*[Title] OR meridia*[Title] OR auricular[Title] OR needl*[Title] OR moxibustion[Title] OR moxa[Title])) AND ((dysmenorr*[Title] OR menstrua* pain[Title] OR period cramp[Title] OR period pain*[Title] OR menstrua* distress[Title] OR period distress[Title] OR menstrua* distress[Title]))
Records retrieved: 275

SinoMed
#1
检索条件 : ("(按摩"[标题:智能] OR "针刺"[标题:智能] OR "推拿"[标题:智能] OR "针灸"[标题:智能] OR " 电针"[标题:智能] OR "穴位"[标题:智能] OR "穴位按摩"[标题:智能] OR "经络"[标题:智能] OR "耳穴"[标题:智能] OR "针刺"[标题:智能] OR "艾灸"[标题:智能] OR "艾草)"[标题:智能]) AND (痛经 OR 月经疼痛 OR 经期痉挛 OR 经期疼痛 OR 经期困扰 OR 月经困扰)
Records retrieved: 2266
#2
检索条件 : ("(按摩"[标题] OR "针刺"[标题] OR "推拿"[标题] OR "针灸"[标题] OR "电针"[标题] OR "穴位 "[标题] OR "穴位按摩"[标题] OR "经络"[标题] OR "耳穴"[标题] OR "针刺"[标题] OR "艾灸"[标题] OR "艾草)"[标题]) AND (痛经 OR 月经疼痛 OR 经期痉挛 OR 经期疼痛 OR 经期困扰 OR 月经困扰)
Records retrieved: 2092

CQVIP
题名=(按摩 OR 针刺 OR 推拿 OR 针灸 OR 电针 OR 穴位 OR 穴位按摩 OR 经络 OR 耳穴 OR 针刺 OR 艾灸 OR 艾草) AND 题名=(痛经 OR 月经疼痛 OR 经期痉挛 OR 经期疼痛 OR 经期困扰 OR 月经困扰)
Records retrieved: 1872

Appendix 2. Excluded studies that were read in full-text and the reasons for exclusion

Study	Original title	Reason for exclusion
**Zhang (2021)**	Effectiveness and Safety of Moxibustion Robots on Primary Dysmenorrhea: A Randomized Controlled Pilot Trial	Both intervention groups used moxibustion (manual moxibustion vs robot moxibustion).
**Youn (2008)**	Effect of Acupuncture Treatment on the Primary Dysmenorrhea: A Study of Single Blind, Sham Acupuncture, Randomized, Controlled Clinical Trial	The full-text was published in Korean.
**Li (2018)**	逆灸法治疗女兵原发性痛经疗效观察及对 PGE、β-EP 水平的调节作用	The study does not have pain outcome measures.
**Zhang (2017)**	针刺三阴交对原发性痛经患者静息态海马功能连接度的影响	The study does not have pain outcome measures.
**Jia (2019)**	隔姜铺灸治疗寒湿凝滞型原发性痛经疗效观察	The control group is usual care.
**Tian (2021)**	Acupuncture for dysmenorrhea of adenomyosis: A randomized controlled trial	The target condition is not primary dysmenorrhea.
**Liu (2019)**	Effect of herb-partitioned moxibustion for primary dysmenorrhea: a randomized clinical trial	The treatment is herb-partitioned moxibustion, which combined herb and moxibustion.
**Bahrami-Taghanaki (2017)**	Effects of acupuncture and mefenamic acid on primary dysmenorrhea	The full-text was published in Iranian.
**Wang (2009)**	Effects of Auricular Acupressure on Menstrual Symptoms and Nitric Oxide for Women with Primary Dysmenorrhea	The study does not have pain outcome measures.
**Yaghobinejad (2017)**	The effect of SP6 moxibustion and acupressure on the severity of systemic symptoms associated with dysmenorrhea	The full-text was published in Iranian.
**Li (2020)**	任脉长蛇灸治疗寒凝血瘀型原发性痛经的疗效观察	The control is treated with traditional acupuncture and moxibustion.
**Chen (2013)**	温针灸治疗寒湿凝滞型原发性痛经的临床研究	The study does not have pain outcome measures.
**Du (2012)**	经前针刺十七椎穴治疗原发性痛经的临床研究	The study does not have pain outcome measures.
**Cong (2011)**	针刺治疗原发性痛经止痛效果观察	The study does not have pain outcome measures.
**Bi (2014)**	Primary dysmenorrhea treated with staging acupoint catgut embedment therapy: a randomized controlled trial	The treatment is staging acupoint catgut embedment therapy.
**Fu (2018)**	Acupoint Application with Daiwenjiu Plaster for Primary Dysmenorrhea of Cold-Dampness Stagnation Syndrome	The treatment is acupoint application with Daiwenjiu plaster.
**Leng (2016)**	铺药隔姜灸治疗原发性痛经 24 例临床观察	The study does not have pain outcome measures.
**Ye (2018)**	温通散神阙穴敷贴治疗寒凝血瘀型原发性痛经的疗效及作用机制研究	The study does not have pain outcome.
**Song (2015)**	透穴埋线治疗原发性痛经的临床观察	The treatment is staging acupoint catgut embedment therapy
**Zhang (2019)**	腹部十字灸治疗原发性痛经（寒凝血瘀型）的临床研究	The treatment is the combination of Chinese herb and moxibustion.
**Zhang (2019)**	穴位埋线治疗原发性痛经（气滞血瘀型）的疗效观察	The treatment is staging acupoint catgut embedment therapy.
**Li (2018)**	Randomized Controlled Trail of Treating Primary Dysmenorrhea with Shaofu-Zhuyu decoction acupoint application	The treatment is Shaofu-Zhuyu decoction acupoint application.
**Li (2017)**	艾灸对原发性痛经症状及睡眠质量的影响	The control is usual care.
**Du (2018)**	Clinical Observation on Acupoint Application of Wenjing Sanhan Decoction on Primary Pysmenorrhea	The treatment is Wenjing Sanhan Decoction by acupoint application.
**Wang (2014)**	穴位埋线治疗寒凝血瘀型原发性痛经临床观察	The treatment is staging acupoint catgut embedment therapy.
**Fan (2020)**	穴位埋线周期疗法治疗原发性痛经的临床研究	The treatment is staging acupoint catgut embedment therapy.
**Tan (2018)**	基于“经络诊察”穴位埋线及点穴治疗原发性痛经的临床研究	The treatment is staging acupoint catgut embedment therapy.
**Zheng (2021)**	穴位埋线治疗原发性痛经的临床观察	The treatment is staging acupoint catgut embedment therapy.
**Yan (2015)**	穴位敷贴法治疗气滞血瘀型原发性痛经	The treatment is staging acupoint catgut embedment therapy.

Appendix 3. Details of quantitative data for network meta-analysis

Code	The first author (year)	Outcomes	Intervention				Comparison			
			Name	N	Mean	SD	Name	N	Mean	SD
1	Chen (2015)	VAS	Acupressure	65	3.50	1.64	Health education	64	3.91	1.40
2	Dincer (2021)	VAS	Acupuncture	34	1.41	1.40	Sham acupuncture	33	1.73	1.23
3	Gao (2015)	VAS	Moxibustion	24	2.32	1.70	Sham moxibustion	20	3.755	2.30
4	Kashefi (2010)	VAS	Acupressure	39	5.67	1.64	Sham acupressure	41	7.04	1.58
5	Kiran (2013)	VAS	Acupuncture	11	0.75	0.63	NSAIDs	24	1.10	1.01
6	Liu (2022)	VAS	Acupuncture	22	3.35	1.50	Sham acupuncture	19	5.39	1.39
7	Mirbagher-Ajorpaz (2011)	VAS	Acupressure	15	1.66	1.98	Sham acupressure	15	4.80	1.37
8	Qorbanalipour (2018)	VAS	Electro-acupuncture	31	3.38	1.85	Acupressure	33	4.01	1.21
9	Shetty (2018)	VAS	Acupuncture	30	2.54	1.78	Waitlist	30	6.83	1.38
10	Sriprasert (2015)	NRS	Acupuncture	27	6.03	2.14	Oral contraceptive pill	25	4.04	2.91
11	Wang (2019)	VAS	Acupuncture	31	3.62	1.49	NSAIDs	31	5.71	1.32
12	Yang (2017)	VAS	Moxibustion	75	2.54	1.41	NSAIDs	72	2.47	1.29
13	Ding (2021)	VAS	Moxibustion	104	5.26	1.01	NSAIDs	104	6.84	1.17
14	Wan (2022)	VAS	Moxibustion	30	2.76	2.12	NSAIDs	30	4.28	2.46
15	Liu (2019)	VAS	Warm needling	32	4.33	4.04	Acupuncture	32	4.13	4.55
16	Liu (2018)	VAS	Moxibustion	13	2.18	1.04	NSAIDs	13	3.92	1.26
17	Ye (2022)	VAS	Moxibustion	37	3.11	1.41	NSAIDs	37	5.22	1.36
18	Wu (2009)	VAS	Moxibustion	32	2.56	1.32	Acupuncture	31	4.74	1.93
19	Tang (2012)	VAS	*Tuina*	30	2.11	1.12	NSAIDs	30	2.35	2.48
20	Chang (2020)	VAS	Acupuncture	45	1.03	0.12	NSAIDs	45	2.06	0.26
21	Zhang (2017)	VAS	Electro-acupuncture	35	1.70	2.28	NSAIDs	35	5.27	3.11
22	Zhang (2020)	VAS	Moxibustion	45	2.31	1.00	NSAIDs	45	4.09	0.90
23	Zhang (2019)	VAS	Moxibustion	30	5.07	1.23	NSAIDs	30	5.92	1.31
24	Zhang (2020)	VAS	Moxibustion	30	2.89	1.32	Acupuncture	30	3.33	1.14
25	Peng (2021)	VAS	Acupuncture	30	3.33	1.03	NSAIDs	30	3.10	1.24
26	Cao (2011)	VAS	Acupuncture	29	2.91	1.62	NSAIDs	30	6.93	1.82
27	Cao (2014)	VAS	Acupuncture	31	3.26	1.49	NSAIDs	31	5.71	1.32
28	Zhu (2020)	VAS	*Tuina*	27	4.96	1.00	Acupuncture	33	5.46	0.96
29	Zhu (2015)	VAS	Acupuncture	33	2.84	0.09	NSAIDs	33	2.83	0.09
30	Li (2014)	VAS	Acupuncture	10	2.80	1.20	Sham acupuncture	10	4.10	1.20
31	Li (2017)	VAS	Auricular therapy	35	3.00	1.60	NSAIDs	35	3.20	1.40
32	Lin (2019)	VAS	Acupuncture	28	1.27	0.18	NSAIDs	31	1.98	0.17
33	Lin (2020)	NRS	Warm needling	55	2.40	1.39	NSAIDs	55	3.52	1.27
34	Lin (2020)	VAS	Warm needling	31	3.02	1.99	NSAIDs	30	3.40	2.69
35	Liang (2021)	VAS	Moxibustion	30	7.56	1.61	NSAIDs	30	7.06	2.67
36	Fan (2014)	VAS	Moxibustion	30	4.21	1.28	Acupuncture	30	4.37	1.56
37	Tang (2015)	VAS	Acupuncture	30	2.53	0.68	NSAIDs	30	3.55	0.63
38	Wen (2021)	VAS	Acupuncture	30	2.87	1.55	NSAIDs	30	2.27	1.23

39	Wang (2019)	VAS	Acupuncture	30	1.40	0.81	NSAIDs	30	1.96	0.88
40	Wang (2018)	VAS	Moxibustion	60	4.40	1.10	NSAIDs	60	5.50	1.20
41	Bai (2018)	VAS	Moxibustion	40	2.53	0.64	NSAIDs	40	6.53	0.98
42	Sheng (2019)	VAS	Acupuncture	31	5.84	3.25	NSAIDs	30	8.08	3.62
43	Shi (2022)	NRS	Scraping therapy	30	1.97	1.75	NSAIDs	30	3.40	2.19
44	Shi (2017)	VAS	Acupuncture	22	3.91	0.53	NSAIDs	22	4.53	0.20
45	Lou (2020)	VAS	Acupressure	28	4.11	1.91	NSAIDs	30	4.57	1.78
46	Zhai (2020)	VAS	Acupressure	39	2.82	2.01	Sham acupressure	36	5.36	4.04
47	Xiao (2016)	VAS	Acupuncture	30	1.90	1.16	NSAIDs	30	2.03	0.93
48	Fan (2021)	VAS	Moxibustion	30	3.37	1.58	NSAIDs	30	3.43	1.59
49	Jia (2017)	VAS	Acupuncture	51	3.06	2.18	NSAIDs	53	3.69	2.19
50	Hao (2018)	VAS	Acupuncture	30	2.51	2.25	NSAIDs	30	3.69	2.73
51	Guo (2021)	VAS	Moxibustion	41	3.79	0.73	NSAIDs	41	4.83	0.68
52	Zhong (2017)	VAS	Warm needling	33	2.29	0.82	NSAIDs	31	5.48	0.81
53	Chen (2011)	VAS	Acupressure	30	3.32	0.78	NSAIDs	30	6.35	0.95
54	Chen (2014)	VAS	Acupuncture	40	1.24	1.34	NSAIDs	40	2.61	2.03
55	Chen (2022)	VAS	Acupuncture	21	4.13	2.01	Waitlist	16	6.25	1.61
56	Han (2015)	VAS	Acupuncture	30	0.20	0.4	Sham acupuncture	30	1.50	0.90
57	Wei (2019)	VAS	Acupuncture	37	2.14	0.73	NSAIDs	38	2.99	0.56

Abbreviations: VAS: VisualAnalogue Scale; NRS: Numerical Rating Scale; NSAIDs: Non-steroidal anti-inflammatory drugs.

Appendix 4. Pairwise comparison for meta-analysis

Study or Subgroup	Moxibustion Mean	SD	Total	NSAIDs Mean	SD	Total	Weight	Std. Mean Difference IV, Random, 95% CI
Bai 2018	2.53	0.64	40	6.53	0.98	40	7.4%	−4.79 [−5.66, −3.91]
Ding 2021	5.26	1.01	104	6.84	1.17	104	8.8%	−1.44 [−1.75, −1.13]
Fan 2021	3.37	1.58	30	3.43	1.59	30	8.4%	−0.04 [−0.54, 0.47]
Guo 2021	3.79	0.73	41	4.83	0.68	41	8.5%	−1.46 [−1.95, −0.97]
Liang 2021	7.56	1.61	30	7.06	2.67	30	8.4%	0.22 [−0.28, 0.73]
Liu 2018	2.18	1.04	13	3.92	1.26	13	7.4%	−1.46 [−2.34, −0.58]
Wan 2022	2.76	2.12	30	4.28	2.46	30	8.4%	−0.65 [−1.17, −0.13]
Wang 2018	4.4	1.1	60	5.5	1.2	60	8.7%	−0.95 [−1.33, −0.57]
Yang 2017	2.54	1.41	75	2.47	1.29	72	8.8%	0.05 [−0.27, 0.37]
Ye 2022	3.11	1.41	37	5.22	1.36	37	8.4%	−1.51 [−2.03, −0.99]
Zhang 2019	5.07	1.23	30	5.92	1.31	30	8.4%	−0.66 [−1.18, −0.14]
Zhang 2020	2.31	1	45	4.09	0.9	45	8.4%	−1.86 [−2.35, −1.36]
Total (95% CI)			535			532	100.0%	−1.17 [−1.71, −0.62]

Heterogeneity: Tau² = 0.86; Chi² = 178.05, df = 11 (P < 0.00001); I² = 94%
Test for overall effect: Z = 4.17 (P < 0.0001)

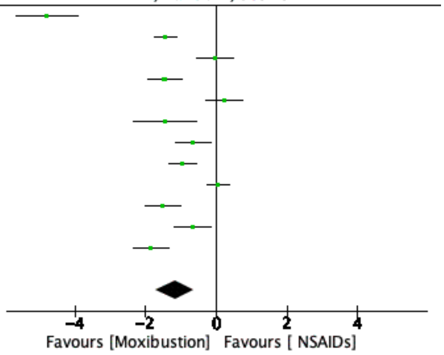

Study or Subgroup	Moxibustion Mean	SD	Total	Acupuncture Mean	SD	Total	Weight	Std. Mean Difference IV, Random, 95% CI
Fan 2014	4.21	1.28	30	4.37	1.56	30	33.7%	−0.11 [−0.62, 0.40]
Wu 2009	2.56	1.32	32	4.74	1.93	31	32.7%	−1.31 [−1.85, −0.76]
Zhang 2020	2.89	1.32	30	3.33	1.14	30	33.6%	−0.35 [−0.86, 0.16]
Total (95% CI)			92			91	100.0%	−0.58 [−1.28, 0.12]

Heterogeneity: Tau² = 0.31; Chi² = 10.80, df = 2 (P = 0.005); I² = 81%
Test for overall effect: Z = 1.63 (P = 0.10)

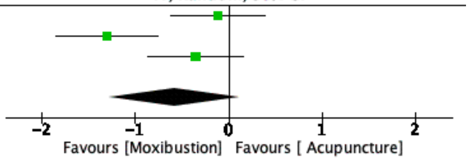

Study or Subgroup	Acupuncture Mean	SD	Total	Waitlist Mean	SD	Total	Weight	Std. Mean Difference IV, Random, 95% CI
Chen 2022	4.13	2.01	21	6.25	1.61	16	50.0%	−1.12 [−1.83, −0.42]
Shetty 2018	2.54	1.78	30	6.83	1.38	30	50.0%	−2.66 [−3.36, −1.95]
Total (95% CI)			51			46	100.0%	−1.89 [−3.40, −0.38]

Heterogeneity: Tau² = 1.05; Chi² = 9.13, df = 1 (P = 0.003); I² = 89%
Test for overall effect: Z = 2.46 (P = 0.01)

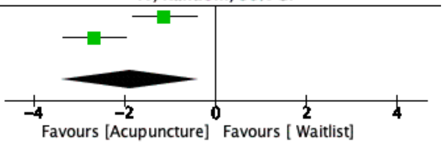

Study or Subgroup	Acupuncture Mean	SD	Total	NSAIDs Mean	SD	Total	Weight	Std. Mean Difference IV, Random, 95% CI
Cao 2011	2.91	1.62	29	6.93	1.82	30	5.1%	−2.30 [−2.97, −1.63]
Cao 2014	3.26	1.49	31	5.71	1.32	31	5.3%	−1.72 [−2.31, −1.13]
Chang 2020	1.03	0.12	45	2.06	0.26	45	4.8%	−5.04 [−5.90, −4.18]
Chen 2014	1.24	1.34	40	2.61	2.03	40	5.4%	−0.79 [−1.24, −0.33]
Hao 2018	2.51	2.25	30	3.69	2.73	30	5.4%	−0.47 [−0.98, 0.05]
Jia 2017	3.06	2.18	51	3.69	2.19	53	5.5%	−0.29 [−0.67, 0.10]
Kiran 2013	0.75	0.63	11	1.1	1.01	24	5.1%	−0.38 [−1.09, 0.34]
Lin 2019	1.27	0.18	28	1.98	0.17	31	4.7%	−4.01 [−4.91, −3.10]
Lou 2020	4.11	1.91	28	4.57	1.78	30	5.4%	−0.25 [−0.76, 0.27]
Peng 2021	3.33	1.03	30	3.1	1.24	30	5.4%	0.20 [−0.31, 0.71]
Sheng 2019	5.84	3.25	31	8.08	3.62	30	5.4%	−0.64 [−1.16, −0.13]
Shi 2017	3.91	0.53	22	4.53	0.2	22	5.1%	−1.52 [−2.20, −0.84]
Tang 2015	2.53	0.68	30	3.55	0.63	30	5.3%	−1.54 [−2.12, −0.96]
Wang 2019	3.62	1.49	31	5.71	1.32	31	5.3%	−1.47 [−2.03, −0.90]
Wang 2019	1.4	0.81	30	1.96	0.88	30	5.4%	−0.65 [−1.17, −0.13]
Wei 2019	2.14	0.73	37	2.99	0.56	38	5.4%	−1.30 [−1.80, −0.80]
Wen 2021	2.87	1.55	30	2.27	1.23	30	5.4%	0.42 [−0.09, 0.94]
Xiao 2016	1.9	1.16	30	2.03	0.93	30	5.4%	−0.12 [−0.63, 0.38]
Zhu 2015	2.84	0.99	33	2.83	0.09	33	5.4%	0.01 [−0.47, 0.50]
Total (95% CI)			597			618	100.0%	−1.11 [−1.58, −0.63]

Heterogeneity: Tau² = 1.04; Chi² = 259.67, df = 18 (P < 0.00001); I² = 93%
Test for overall effect: Z = 4.53 (P < 0.00001)

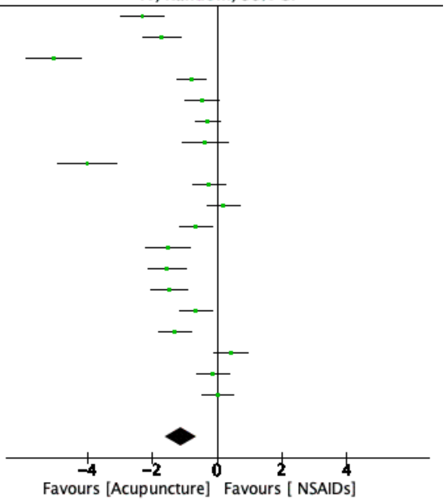

Study or Subgroup	Acupuncture Mean	SD	Total	Sham acupuncture Mean	SD	Total	Weight	Std. Mean Difference IV, Random, 95% CI
Dincer 2021	1.41	1.4	34	1.73	1.23	33	27.6%	−0.24 [−0.72, 0.24]
Han 2015	0.2	0.4	30	1.5	0.9	30	26.0%	−1.84 [−2.45, −1.23]
Li 2014	2.8	1.2	10	4.1	1.2	10	21.5%	−1.04 [−1.98, −0.09]
Liu 2022	3.35	1.5	22	5.39	1.39	19	25.0%	−1.38 [−2.07, −0.69]
Total (95% CI)			96			92	100.0%	−1.11 [−1.92, −0.31]

Heterogeneity: Tau² = 0.55; Chi² = 18.08, df = 3 (P = 0.0004); I² = 83%
Test for overall effect: Z = 2.71 (P = 0.007)

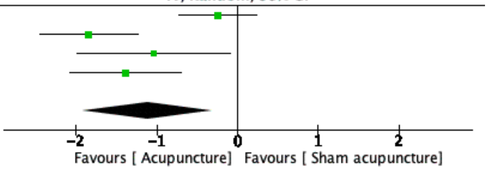

Study or Subgroup	Acupressure Mean	Acupressure SD	Acupressure Total	Sham acupressure Mean	Sham acupressure SD	Sham acupressure Total	Weight	Std. Mean Difference IV, Random, 95% CI
Mirbagher-Ajorpaz 2011	1.66	1.98	15	4.8	1.37	15	43.1%	−1.79 [−2.66, −0.93]
Zhai 2020	2.82	2.01	39	5.36	4.04	36	56.9%	−0.80 [−1.27, −0.33]
Total (95% CI)			54			51	100.0%	−1.23 [−2.19, −0.26]

Heterogeneity: $Tau^2 = 0.37$; $Chi^2 = 3.93$, $df = 1$ ($P = 0.05$); $I^2 = 75\%$
Test for overall effect: $Z = 2.49$ ($P = 0.01$)

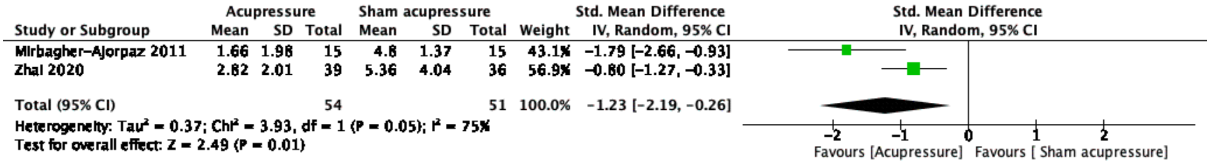

Study or Subgroup	Acupressure Mean	Acupressure SD	Acupressure Total	Sham acupressure Mean	Sham acupressure SD	Sham acupressure Total	Weight	Std. Mean Difference IV, Random, 95% CI
Chen 2011	3.32	0.78	30	6.35	0.95	30	49.1%	−3.44 [−4.25, −2.63]
Kashefi 2010	5.67	1.64	39	7.04	1.58	41	50.9%	−0.84 [−1.30, −0.38]
Total (95% CI)			69			71	100.0%	−2.12 [−4.66, 0.43]

Heterogeneity: $Tau^2 = 3.26$; $Chi^2 = 29.74$, $df = 1$ ($P < 0.00001$); $I^2 = 97\%$
Test for overall effect: $Z = 1.63$ ($P = 0.10$)

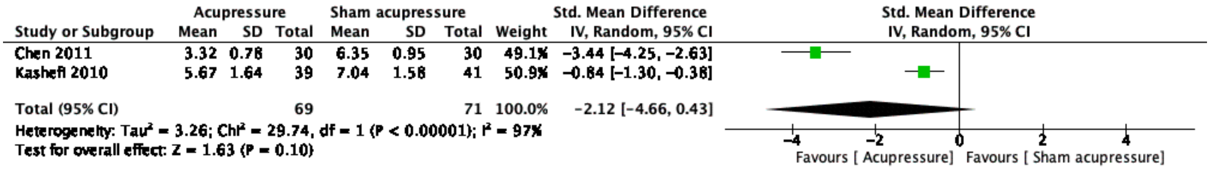

Study or Subgroup	Warm needling Mean	Warm needling SD	Warm needling Total	NSAIDs Mean	NSAIDs SD	NSAIDs Total	Weight	Std. Mean Difference IV, Random, 95% CI
Lin 2020	2.4	1.39	55	3.52	1.27	55	34.2%	−0.84 [−1.23, −0.45]
Lin 2020	3.02	1.99	31	3.4	2.69	30	33.8%	−0.16 [−0.66, 0.34]
Zhong 2017	2.29	0.82	33	5.48	0.81	31	32.0%	−3.87 [−4.71, −3.02]
Total (95% CI)			119			116	100.0%	−1.58 [−3.23, 0.08]

Heterogeneity: $Tau^2 = 2.03$; $Chi^2 = 55.30$, $df = 2$ ($P < 0.00001$); $I^2 = 96\%$
Test for overall effect: $Z = 1.87$ ($P = 0.06$)

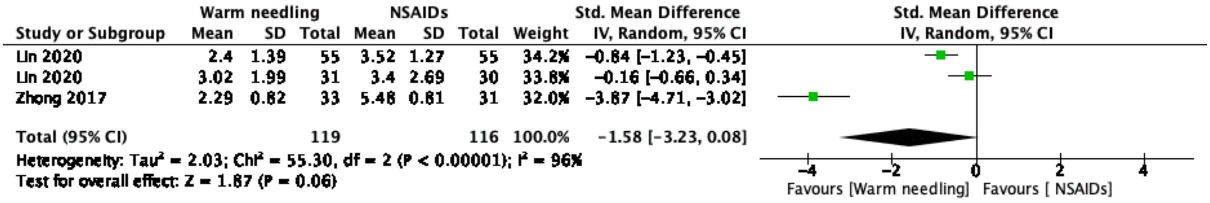

Appendix 5. The results of local inconsistency test

Side	Direct		Indirect		Difference			tau
	Coef.	Std. Err.	Coef.	Std. Err.	Coef.	Std. Err.	P>|z|	
Acupressure-Health education	.	.	.	.	.	.	.	.
Acupressure- NSAIDs	1.794	0.912	1.052	1.173	0.742	1.485	0.617	1.242
Acupressure- Electro-acupuncture	-0.401	1.270	0.339	1.496	-0.740	1.963	0.706	1.245
Acupressure- Sham acupressure	1.127	0.741	1.566	1.589	-0.438	1.754	0.803	1.245
Sham acupuncture-Acupuncture *	-1.144	0.732	1.995	365.238	-3.139	365.239	0.993	1.230
Acupuncture- Moxibustion	-0.705	0.896	0.029	0.453	-0.735	1.004	0.464	1.238
Acupuncture- NSAIDs	1.161	0.301	0.723	0.676	0.437	0.740	0.554	1.241
Acupuncture- Waitlist *	1.888	0.906	1.039	447.759	0.849	447.760	0.998	1.230
Acupuncture-Oral contraceptive pill *	-0.771	1.263	-1.621	632.806	0.850	632.807	0.999	1.230
Acupuncture-*Tuina*	-0.504	1.263	1.001	1.292	-1.505	1.808	0.405	1.236
Acupuncture-Warm needling	0.045	1.270	-0.491	0.792	0.537	1.496	0.720	1.245
Acupuncture-Sham acupressure	1.034	1.336	0.595	1.136	0.438	1.754	0.803	1.245
Moxibustion-NSAIDs	1.119	0.352	1.854	0.940	-0.735	1.004	0.464	1.238
Moxibustion-Sham moxibustion *	0.706	1.269	0.096	632.048	0.609	632.049	0.999	1.230
NSAIDs-Electro-acupuncture	-1.294	1.272	-2.034	1.494	0.740	1.963	0.706	1.245
NSAIDs-*Tuina*	-0.122	1.262	-1.628	1.293	1.505	1.808	0.405	1.236
NSAIDs-Auricular therapy *	-0.131	1.253	-3.169	632.423	3.037	632.424	0.996	1.230
NSAIDs-Warm needling	-1.561	0.740	-1.024	1.300	-0.537	1.496	0.720	1.245
NSAIDs-Scraping therapy *	-0.711	1.259	-3.740	632.939	3.029	632.940	0.996	1.230

Appendix 6. Sensitivity analysis

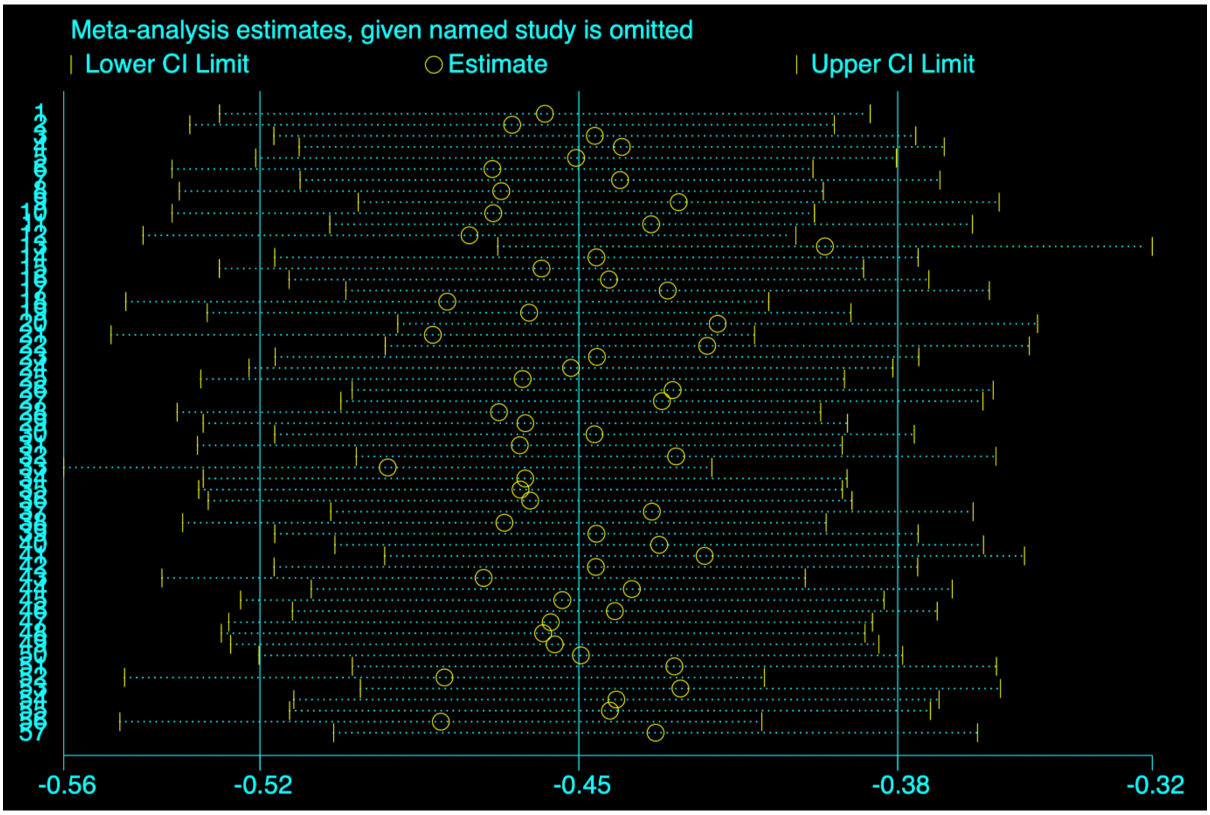

Appendix 7. Adverse event of the interventions

No.	Author (yr)	Intervention	Adverse events	Comparison	Adverse events
1	Kashefi (2010)	Acupressure	NR	Sham acupressure	Dropout due to adverse event (n=2)
2	Kiran (2013)	Acupuncture	NR	NSAIDs	Pain-killer effect of medication faded away with repetitive use in some cases
3	Sriprasert (2015)	Acupuncture	Minor bleeding at acupuncture points (n=15), headache or myalgia (n=4), fever (n=1)	NSAIDs	Abnormal vaginal bleeding (n=9), headache or myalgia (n=5), weight gain (n=3), nausea or vomiting (n=2), breast engorgement (=2)
4	Wang (2019)	Acupuncture	Hematoma occurred during or after treatment (n=7)	NSAIDs	Gastrointestinal irritation, such as nausea, anorexia, heartburn, bloating (n=17)
5	Chang (2020)	Acupuncture	Nausea (n=1)	NSAIDs	Nausea (n=1), dyspepsia (n=1), heartburn (n=2), anorexia (n=3)
6	Zhang (2020)	Moxibustion	NR	NSAIDs	Hypermenorrhea (n=2), stomach upset (n=2)
7	Peng (2021)	Acupuncture	Hematoma (n=1)	NSAIDs	Gastrointestinal irritation (n=2)
8	Cao (2011)	Acupuncture	NR	NSAIDs	Rash (n=1), loss of appetite (n=2)
9	Cao (2014)	Acupuncture	Hematoma (n=7)	NSAIDs	Gastrointestinal symptom: nausea, anorexia, heartburn, bloating (n=17)
10	Lin (2019)	Acupuncture	Minor bleeding at acupuncture points (n=2)	Oral contraceptive pill	NR
11	Lin (2020)	Warm needling	Minor bleeding at acupuncture points (n=4)	NSAIDs	NR
12	Wang (2019)	Acupuncture	NR	NSAIDs	Gastrointestinal symptom: nausea, mild indigestion and a burning stomach (n=3)
13	Sheng (2019)	Acupuncture	Minor bleeding at acupuncture points (n=1), sticking of needle (n=3)	NSAIDs	Gastrointestinal symptom: nausea, stomach burning sensation, mild indigestion (n=2), dizziness and tinnitus (n=1)
14	Shi (2022)	Scraping therapy	NR	NSAIDs	Mild gastrointestinal reaction (n=2)
15	Lou (2020)	Acupressure	Nervousness in the first treatment session	NSAIDs	NR
16	Zhai (2020)	Acupressure	NR	Sham acupressure	Dropout due to severe pain (n=3)
17	Xiao (2016)	Acupuncture	Minor bleeding at acupuncture points	NSAIDs	Gastrointestinal symptom: nausea and vomiting (n=15), dizziness (n=3)
18	Jia (2017)	Acupuncture	NR	NSAIDs	Gastrointestinal reaction (n=1)
19	Wei (2019)	Acupuncture	NR	Western medicine	Gastrointestinal symptom: dyspepsia (n=1), dizziness (n=1)

Appendix 8. Summary of Findings (SoF) table based on GRADEpro evaluation

Outcomes	Anticipated absolute effects* (95% CI)		Relative effect (95% CI)	No of participants (studies)	Certainty of the evidence (GRADE)	Comments
	Risk with control	Risk with intervention				
**Acupressure compared to health education on primary dysmenorrhea pain**						
Pain	-	SMD 0.27 SD lower (0.62 lower to 0.08 higher)	-	129 (1 RCT)	⨁⨁◯◯ Low	①②
**Acupuncture compared to placebo on primary dysmenorrhea pain**						
Pain	-	SMD 1.41 SD lower (2.1 lower to 0.72 lower)	-	121 (3 RCTs)	⨁⨁⨁⨁ High	①
**Moxibustion compared to placebo on primary dysmenorrhea pain**						
Pain	-	SMD 0.72 SD lower (1.33 lower to 0.11 lower)	-	44 (1 RCT)	⨁⨁◯◯ Low	①②③
**Acupressure compared to placebo on primary dysmenorrhea pain**						
Pain	-	SMD 0.83 SD lower (1.33 lower to 0.34 lower)	-	252 (4 RCTs)	⨁⨁⨁◯ Moderate	①③
**Acupuncture compared to western medicine on primary dysmenorrhea pain**						
Pain	-	SMD 0.38 SD lower (1.1 lower to 0.34 higher)	-	1209 (19 RCTs)	⨁⨁◯◯ Low	③④
**Electroacupuncture compared to acupressure on primary dysmenorrhea pain**						
Pain	-	SMD 0.41 SD lower (0.9 lower to 0.09 higher)	-	64 (1 RCT)	⨁⨁◯◯ Low	①②
**Electroacupuncture compared to western medicine on primary dysmenorrhea pain**						
Pain	-	SMD 1.31 SD lower (1.83 lower to 0.79 lower)	-	70 (1 RCT)	⨁⨁⨁⨁ High	①
**Acupuncture compared to waitlist on primary dysmenorrhea pain**						
Pain	-	SMD 2.69 SD lower (3.4 lower to 1.99 lower)	-	97 (2 RCTs)	⨁⨁◯◯ Low	①②③④
**Moxibustion compared to NSAIDs on primary dysmenorrhea pain**						
Pain	-	SMD 0.05 SD higher (0.27 lower to 0.38 higher)	-	1067 (12 RCTs)	⨁⨁⨁◯ Moderate	③
**Moxibustion compared to acupuncture on primary dysmenorrhea pain**						
Pain	-	SMD 1.32 SD lower (1.87 lower to 0.78 lower)	-	183 (3 RCTs)	⨁⨁⨁◯ Moderate	①②③
***Tunia* compared to NSAIDs on primary dysmenorrhea pain**						
Pain	-	SMD 0.12 SD lower (0.63 lower to 0.38 higher)	-	60 (1 RCT)	⨁⨁◯◯ Low	①②
***Tunia* compared to acupuncture on primary dysmenorrhea pain**						
Pain	-	SMD 0.51 SD lower (1.03 lower to 0.01 higher)	-	60 (1 RCT)	⨁⨁⨁◯ Moderate	①②
**Auricular therapy compared to NSAIDs on primary dysmenorrhea pain**						
Pain	-	SMD 0.13 SD lower (0.6 lower to 0.34 higher)	-	70 (1 RCT)	⨁◯◯◯ Very low	①②④
**Warm needling compared to acupuncture on primary dysmenorrhea pain**						
Pain	-	SMD 0.05 SD higher (0.44 lower to 0.54 higher)	-	64 (1 RCT)	⨁⨁⨁◯ Moderate	①②
**Warm needling compared to NSAIDs on primary dysmenorrhea pain**						
Pain	-	SMD 0.84 SD lower (1.23 lower to 0.45 lower)	-	235 (3 RCTs)	⨁◯◯◯ Very low	①②③
**Scraping therapy compared to NSAIDs on primary dysmenorrhea pain**						
Pain	-	SMD 0.72 SD lower (1.24 lower to 0.2 lower)	-	60 (1 RCT)	⨁⨁⨁◯ Moderate	①②

Appendix 8. Summary of Findings (SoF) table based on GRADEpro evaluation

Outcomes	**Anticipated absolute effects*** (95% CI)		Relative effect (95% CI)	No of participants (studies)	Certainty of the evidence (GRADE)	Comments
	**Risk with control**	**Risk with intervention**				
**Acupressure compared to NSAIDs on primary dysmenorrhea pain**						
Pain	-	SMD 0.25 SD lower (0.77 lower to 0.27 higher)	-	118 (2 RCTs)	⊕◯◯◯ Very low	①②③
**Acupressure compared to oal contraceptive pillon primary dysmenorrhea pain**						
Pain	-	SMD 0.77 higher (0.06 higher to 1.48 higher)	-	52 (1 RCT)	⊕⊕◯◯ Low	①②

***The risk in the intervention group** (and its 95% confidence interval) is based on the assumed risk in the comparison group and the **relative effect** of the intervention (and its 95% CI).
CI: confidence interval; **SMD:** standardised mean difference

GRADE Working Group grades of evidence
High certainty: we are very confident that the true effect lies close to that of the estimate of the effect.
Moderate certainty: we are moderately confident in the effect estimate: the true effect is likely to be close to the estimate of the effect, but there is a possibility that it is substantially different.
Low certainty: our confidence in the effect estimate is limited: the true effect may be substantially different from the estimate of the effect.
Very low certainty: we have very little confidence in the effect estimate: the true effect is likely to be substantially different from the estimate of effect.

Remarks of the comments
① The sample size from the included studies does not meet the optimal sample size ($n < 400$)
② The 95% confidence interval crosses the invalid line
③ The heterogeneity among the studies in meta-analysis was larger ($p < 0.1$, $I^2 > 30\%$);
④ The included studies have a large bias in methodology (e.g. randomization, allocation concealment, blinding, reporting, and conflicts of interest);

Appendix 9. Funnel plot and egger test

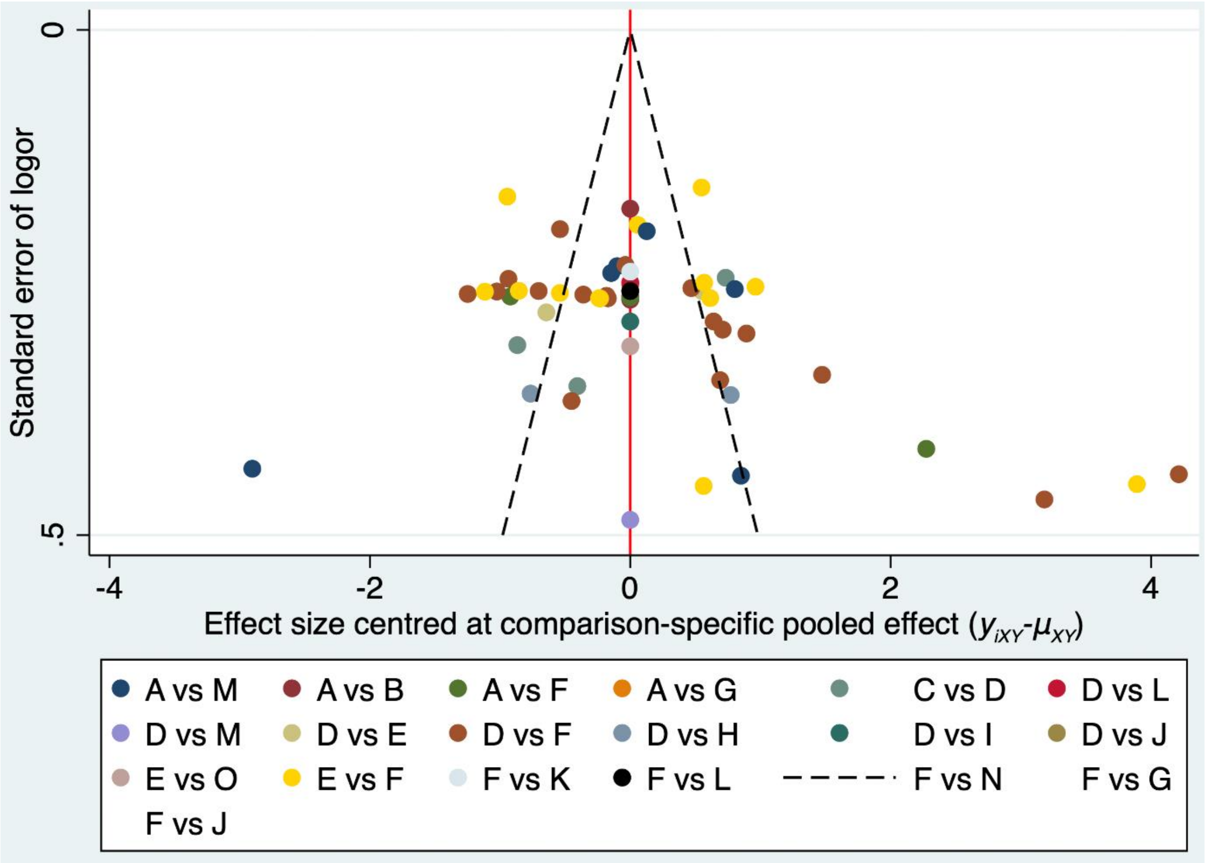

Supplement: Supplementary file 1 [file Presentation_1.pdf]
